# Identification of molecular signatures specific for distinct cranial sensory ganglia in the developing chick

**DOI:** 10.1186/s13064-016-0057-y

**Published:** 2016-01-27

**Authors:** Cedric Patthey, Harry Clifford, Wilfried Haerty, Chris P. Ponting, Sebastian M. Shimeld, Jo Begbie

**Affiliations:** Department of Physiology, Anatomy and Genetics, University of Oxford, Oxford, UK; Department of Zoology, University of Oxford, Oxford, UK; MRC Functional Genomics, University of Oxford, Oxford, UK; Umeå Center for Molecular Medicine, Umeå University, Umeå, Sweden

**Keywords:** Cranial sensory ganglia, Viscerosensory neuron, Somatosensory neuron, Cell type markers, Chicken, FACS, Expression profiling

## Abstract

**Background:**

The cranial sensory ganglia represent populations of neurons with distinct functions, or sensory modalities. The production of individual ganglia from distinct neurogenic placodes with different developmental pathways provides a powerful model to investigate the acquisition of specific sensory modalities. To date there is a limited range of gene markers available to examine the molecular pathways underlying this process.

**Results:**

Transcriptional profiles were generated for populations of differentiated neurons purified from distinct cranial sensory ganglia using microdissection in embryonic chicken followed by FAC-sorting and RNAseq. Whole transcriptome analysis confirmed the division into somato- versus viscerosensory neurons, with additional evidence for subdivision of the somatic class into general and special somatosensory neurons. Cross-comparison of distinct ganglia transcriptomes identified a total of 134 markers, 113 of which are novel, which can be used to distinguish trigeminal, vestibulo-acoustic and epibranchial neuronal populations. In situ hybridisation analysis provided validation for 20/26 tested markers, and showed related expression in the target region of the hindbrain in many cases.

**Conclusions:**

One hundred thirty-four high-confidence markers have been identified for placode-derived cranial sensory ganglia which can now be used to address the acquisition of specific cranial sensory modalities.

**Electronic supplementary material:**

The online version of this article (doi:10.1186/s13064-016-0057-y) contains supplementary material, which is available to authorized users.

## Background

The sensory nervous system is fundamental to perception of our body’s external and internal environments. It is generally accepted that distinct types of sensation are mediated by neurons specialised in responding to specific stimuli, raising questions relating to how these distinct groups of neurons differ, both at the level of physiological function, and at the level of the acquisition of specific phenotypes during development [1]. To this end, recent publications have outlined transcriptome analysis of sensory neurons in the trunk, identifying specific subsets of somatosensory neurons [2, 3]. However, these studies provide molecular signatures specifically for trunk somatosensory neurons, and do not encompass the many other sensory modalities conveyed by cranial sensory neurons [4]. Our aim was to develop a resource which would address the paucity of markers known to distinguish between neurons characteristic of distinct cranial sensory ganglia.

**Fig. 1:**
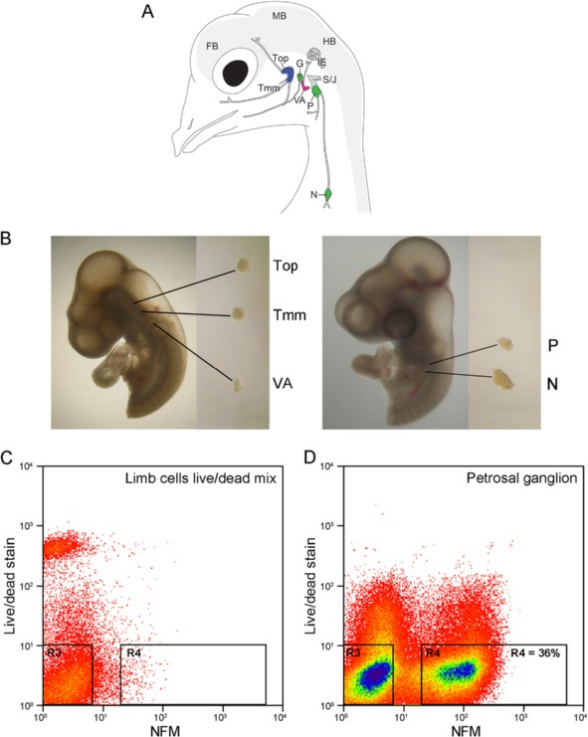
Isolation of embryonic chick placode-derived cranial sensory neurons by dissection and FACS. **A** Schematic of cranial sensory ganglia in embryonic day 12 (HH38) chick (adapted from [6]). The ganglia are labelled: trigeminal ganglion as two separate lobes (Top: ophthalmic; Tmm: maxillomandibular); vestibulo-acoustic ganglion (VA); epibranchial series as three separate ganglia (G: geniculate; P: petrosal; N: nodose). Also labelled are the neural crest-derived superior– jugular ganglionic complex (S/J); the inner ear (IE); and forebrain (FB); midbrain (MB) and hindbrain (HB) of the CNS. Colours indicate the sensory modality of ganglion: blue: general somatosensory; magenta: special somatosensory; green: viscerosensory. **B** Representative dissections of cranial sensory ganglia: Top, Tmm and VA at HH18, and P and N at HH23. **C**, **D** Representative FACS plots of cells stained for live/dead stain and NFM. **C** Control cell population: limb bud cells devoid of neurons, containing 50 % dead cells. **D** Petrosal ganglion cell population: 36 % of cells are NFM positive and dead cell marker negative

The cranial sensory ganglia can be categorised as having distinct sensory modalities according to the function of their associated cranial nerve (Fig. 1A). The trigeminal ganglion (which can be subdivided into ophthalmic and maxillomandibular), associated with cranial nerve V, is considered most similar to the sensory dorsal root ganglia (DRG) in the trunk, being involved in touch, pain and temperature sensation. The vestibulo-acoustic ganglion, associated with cranial nerve VIII, innervates the inner ear structures involved in balance and hearing. The epibranchial ganglia, individually called geniculate, associated with cranial nerve VII; petrosal, associated with cranial nerve IX; and nodose, associated with cranial nerve X, are involved in sensing chemicals such as tastants, digestive catabolites, and blood gas levels, in addition to sensing pressure changes in blood vessels. The cranial sensory modalities thus correspond to somatosensation, which is further subdivided into general somatosensory (trigeminal) and special somatosensory (vestibulo-acoustic), and viscerosensation (geniculate, petrosal and nodose). Our study is focused on the cranial sensory ganglia of chicken (Fig. 1A), but their organisation and function are well conserved across vertebrates [5].

The development of cranial sensory ganglia remains less well studied than that of the DRG, possibly due to their perceived complexity. Compared with the DRG, which all develop exclusively from neural crest, each individual cranial sensory ganglion develops from a distinct neurogenic placode with some neural crest contribution to the proximal cranial sensory ganglia: the proximal region of the trigeminal ganglion, small numbers of neurons in the geniculate and vestibulo-acoustic ganglia, and the entirety of the superior/jugular ganglia associated with cranial nerves IX and X [6–8]. As each different neurogenic placode utilises a distinct developmental path, they can be used to understand the acquisition of different, specific sensory modalities. Experiments addressing placode fate switching through in vivo transplantation and in vitro pathway manipulation, show that the trigeminal (somatosensory) and nodose (viscerosensory) placode are fate-restricted once neurogenesis begins [7, 9, 10]. In the mouse, expression of the transcription factor *Phox2b* underpins the fate choice between these two sensory modalities [11]. However, only a limited range of markers exist that can be used to investigate this cell type decision, and no markers are currently available to distinguish between general and special somatosensory modalities. To extend experimental analysis of cranial sensory ganglia development further, we require a broad range of markers that distinguish between differentiated neurons of different phenotypes.

Even neurons of the same sensory modality can differ depending on whether they derive from neural crest or placode. Analysis of the *Scn10a* gene promoter in the mouse has shown that a specific fragment recapitulates endogenous expression of the product Na_v_1.8 in neural crest-derived but not placode-derived cranial sensory neurons [12]. Furthermore, nociceptive C-fibre sensory neurons innervating the lung are phenotypically distinct depending on whether they are neural crest- or placode-derived [13]. These observations reinforce the importance of producing markers that are specific for placode-derived cranial sensory neurons.

The timing and localisation of distinct placode-derived cranial sensory ganglion development have been carefully documented in the chicken [6, 9, 10, 14–19]. Here we take advantage of our knowledge of the development of the chicken system, combined with genome-wide expression profiling, to characterise ganglion-specific populations of placode-derived sensory neurons at early stages of differentiation. We present RNA-seq data generated from embryonic neurons purified from five distinct cranial sensory ganglia (namely trigeminal maxillomandibular; trigeminal ophthalmic; vestibulo-acoustic; petrosal; and nodose ganglia) separated by dissection and fluorescence-activated cell sorting (FACS). Using this gene expression data, we provide an objective and comprehensive classification of distinct populations of cranial sensory neurons. Whole transcriptome analysis confirms the dichotomy of somatosensory (somatic) versus viscerosensory (visceral) neurons, but additionally provides molecular evidence for the subdivision of the somatosensory neurons into general and special somatosensory neurons as previously described based on anatomy [11, 20]. Cross-comparison of distinct ganglia transcriptomes identifies a total of 134 markers, 113 of them novel, which can be used to distinguish trigeminal, vestibulo-acoustic and epibranchial neuronal populations. We confirm expression of 20 of these specific markers in the specific cranial sensory ganglia by in situ hybridization. Taken together, our data provides molecular signatures for distinct cranial sensory neuronal populations.

## Results

### Transcriptional profile analysis of cranial sensory ganglia placode-derived neurons

In all vertebrates the cranial sensory ganglia are segregated according to sensory function. In the chicken, the stereotypical localisation of the ganglia (Fig. 1A) and our detailed understanding of the timing of their development [6, 14, 18, 19, 21] make it possible to dissect the ganglia separately in order to establish expression profiles of distinct populations of developing sensory neurons. We took advantage of this to harvest the trigeminal (maxillomandibular and ophthalmic), vestibulo-acoustic, nodose and petrosal ganglia from Hamburger-Hamilton stage 18 (HH18) [22] (both trigeminal and vestibulo-acoustic) or HH23 (nodose and petrosal) chicken embryos (Fig. 1B). Collection at these embryonic stages allowed us to compensate for differences in the timing of ganglion development, thus ensuring the neurons would be investigated at a similar stage of differentiation [7, 14, 18, 19]. Furthermore, these specific timings meant that the population of collected neurons exclusively contained placode-derived neurons. This was of particular importance for the trigeminal ganglion where neural crest-derived neurons contribute directly to the ganglion at later stages, rather than forming separate ganglia [6, 8]. We confirmed that we could avoid neural crest-derived neuron contamination of our trigeminal samples in a separate experiment, specifically labelling neural crest cells with GFP and showing that these did not contribute to the neuronal pool at HH18 (Additional file 1: Figure S1). The trigeminal ganglion was collected as two separate lobes (maxillomandibular and ophthalmic) because these arise from distinct placodes with individual characteristics [14, 18, 23], and further exist as two separate ganglia in more basal vertebrates [5, 24].

In order to profile the transcriptomes of differentiated neurons rather than non-neural cell types or neural progenitors we isolated all cells positive for neurofilament medium polypeptide (NFM) antibody staining. To this aim we adapted transcription factor FACS (tfFACS) [25], a method that allows sorting of cells with antibodies raised towards intracellular epitopes. Briefly, freshly dissected ganglia were quickly dissociated to single cells and fixed, thereby freezing the cells in their transcriptional state. The cells were gently permeabilised and subjected to NFM immunostaining, followed by FACS. Prior to fixation the cells were treated with a live/dead stain, with the gate for live/dead cells set using a control limb bud sample containing 50 % of cells killed by heat-shock (Fig. 1C). The extracted NFM positive (NFM+) neurons represented 13–41 % of the total cell population, while dead cells (4–9 % of total) were excluded (Fig. 1D). A total of 20,000 to 290,000 NFM+ neurons per sample were collected by FACS and 50-200 ng of high quality total RNA extracted (Additional file 2). Following this, RNA-sequencing returned a mean of ~77 million (~30–116 million) 100 bp reads for each of three replicates, of which an average of 88 ± 6 % mapped to the genome assembly. An average of 11,800 (10,897–12,190) genes per sample were expressed at an appreciable level (>0.3 read per kilobase per million mapped reads (RPKM), Additional file 3).

**Fig. 2:**
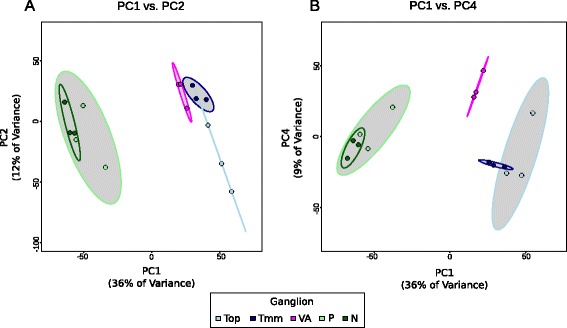
Principal Component Analysis of transcriptome-wide data displaying ganglion-specific clustering. **A** A plot of the first two principal components from analysis on all variance-stabilized gene expressions, coloured by ganglion. **B** Plot of the first and fourth principal components from analysis as in (**A**). The grey ellipses represent 90 % confidence intervals for groupings

Principal Component Analysis (PCA) of gene expression across all samples revealed two distinct, unambiguous clusters indicative of two distinct categories of cranial sensory ganglia captured by the first principal component (Fig. 2A). None of the principal components significantly correlated with RNA integrity measurements (RIN values), RNA yield or sequencing depth (Additional file 2; Additional file 4: Figure S2A, B, C). The clusters reflected the known segregation of viscerosensory neurons (nodose and petrosal) and somatosensory neurons (vestibulo-acoustic and trigeminal) [11]. However, the two clusters also reflected the different embryonic stages of dissection. To test levels of neuronal maturation, we examined expression levels of six known markers of differentiated neurons (*ELAVL4 (HUD), ISL1, MYT1, NEUROD1, RBFOX3 (NEUN)* and *TUBB3 (Tuj1)*)*.* Levels of expression were similar across the two sets of samples, supporting the hypothesis that the clusters reflect sensory phenotype rather than maturation differences (Additional file 5: Figure S3).

The projections of the samples on PC1 and PC2 also show separation of the somatosensory cluster into a trigeminal cluster and a vestibulo-acoustic cluster (Fig. 2A): a conclusion further strengthened by analysis of the other principal components (Fig. 2B). This supports the segregation of these ganglia into general (trigeminal) and special (vestibulo-acoustic) somatosensory ganglia, terminology which has been applied largely based on the anatomy of their central projections with less known about the molecular basis [20]. Thus, our transcriptome-wide analysis supports a clear separation of cranial sensory ganglia into viscerosensory (nodose/petrosal) and somatosensory modalities (trigeminal/vestibulo-acoustic) with further subdivision of the latter into general (trigeminal) and special (vestibulo-acoustic) somatosensory modalities.

**Fig. 3:**
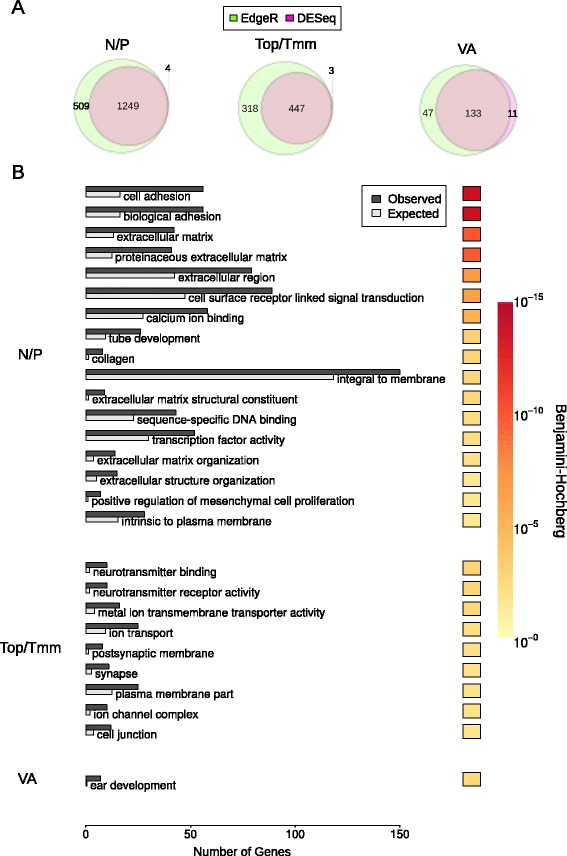
Genes differentially expressed in Nodose and Petrosal (N/P), Trigeminal ophthalmic and maxillomandibular (Top/Tmm) and Vestibulo-acoustic (VA) ganglia. **A** Number of differentially expressed genes (q < 0.05) reported by DESeq and EdgeR. **B** Significant Gene Ontology (q < 0.05) enrichment for differentially expressed genes reported by both DESeq and EdgeR. Redundant GO terms were removed using REVIGO. The Hinton plot displays the FDR (Benjamini and Hochberg) corrected q-value

To identify ganglion-specific gene markers, differentially expressed genes were determined using both DESeq and EdgeR algorithms [26, 27], with the resultant intersect taken to ensure a robust selection. All combinations of ganglia were tested, and differentially expressed genes from both sets of analysis are available as Additional file 6. We were particularly interested in differential expression that reflected the PCA separation of the ganglia into three clusters. Accordingly our analysis identified higher expression of 1249 genes in nodose/petrosal; 447 in trigeminal and 133 in vestibulo-acoustic (Fig. 3A). The numbers of up-regulated genes for other combinations of ganglia were: nodose: 169; petrosal: 7; trigeminal maxillomandibular: 60; trigeminal ophthalmic: 107; and trigeminal maxillomandibular/trigeminal ophthalmic/vestibulo-acoustic: 708 (Additional file 7: Figure S4A, Additional file 6). No differentially expressed genes were found in the remaining combinations of ganglia. Gene ontology (GO) term analysis showed that each cluster of ganglia was characterised by enrichment of a distinct set of GO categories. The nodose/petrosal grouping showed the broadest spread of categories, with adhesion and membrane proteins being particularly enriched (Fig. 3B). The trigeminal grouping was the most overtly neuronal with neurotransmitter and ion transport activity terms enriched (Fig. 3B). Satisfyingly, the significant GO category for the vestibulo-acoustic ganglion, which is associated with the ear, was ear development (Fig. 3B). GO terms enrichments for other combinations of ganglia are listed in Additional file 7: Figure S4B.

### Identification of high-confidence markers for specific cranial sensory ganglia

**Fig. 4:**
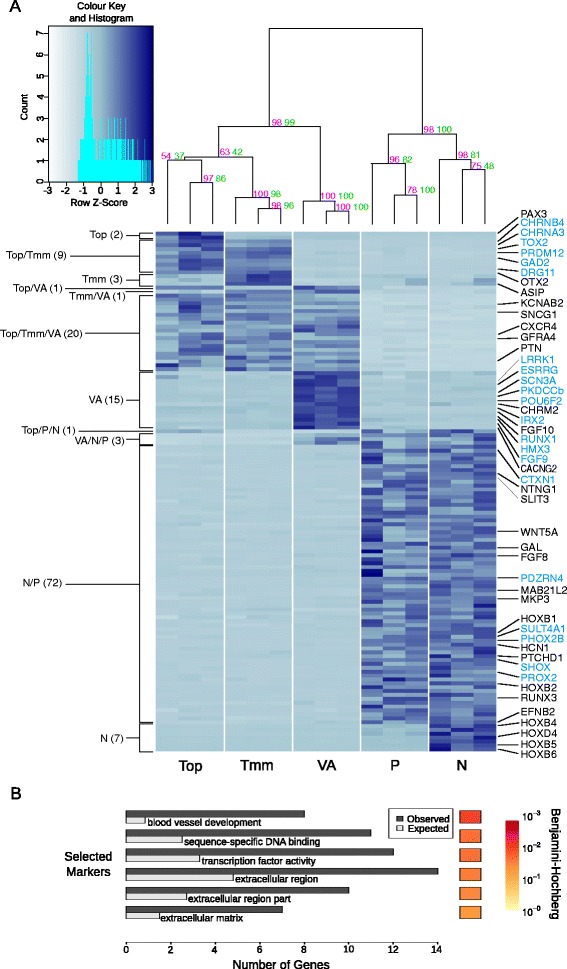
134 high confidence ganglion-specific markers. **A** Variance stabilized gene expression of all markers in ganglia or groups of ganglia. Numbers of ganglion-specific markers are indicated on the left. Genes of interest (*black text*) including those selected for validation by in-situ hybridization (*blue text*) are shown on the right. The dendogram displays the hierarchical clustering of gene expression into ganglia and groups of ganglia. The magenta and green values on each node represent the Approximately Unbiased P-value and the Bootstrap Probability value respectively. **B** Significant Gene Ontology enrichment (q < 0.05) for all markers. Redundant GO terms were removed using REVIGO. The Hinton plot displays the FDR (Benjamini and Hochberg) corrected q-value

Genes identified as being differentially expressed using both DESeq and EdgeR were subjected to stringent selection criteria based on expression level, fold-change, and statistical significance (see Material and Methods) to generate an unbiased panel of genes that best represent individual ganglia and combinations of ganglia (Fig. 4A; Additional file 8). The hierarchical clustering of the resultant 134 markers (Fig. 4A) reflected the division of the cranial sensory ganglia demonstrated by PCA (Fig. 2). The traditional division was represented by 20 markers of the somatosensory ganglia (trigeminal/vestibulo-acoustic) and 72 markers of the viscerosensory ganglia (nodose/petrosal) were found. In addition, we found 9 markers specific for the general somatosensory (trigeminal) and 15 markers for the special somatosensory ganglia (vestibulo-acoustic).

The validity of the marker sets was confirmed by considering genes whose expression has been shown previously to be restricted to specific cranial sensory ganglia. Thus trigeminal ophthalmic expressed *PAX3* [10, 28, 29]; trigeminal maxillomandibular/ophthalmic ganglia expressed *DRG11* [30–32]; and nodose/petrosal ganglia expressed *PHOX2B* [11, 21, 33–35]. Our panel of markers included 7 genes expressed in the nodose ganglion but not in the petrosal ganglion. These included the HOX genes *HOXB4*, −*D4*, −*B5*, and *-B6* (Fig. 4A) reflecting the well-known distribution of HOX gene expression along the rostro-caudal axis. In line with this, the more anteriorly expressed HOX genes, *HOXB1* and *–B2* were included in the nodose/petrosal grouping (Fig. 4A).

Surprisingly, *POU4F1/BRN3A*, a well-known marker of somatosensory neurons in mammals [11, 36], was not among our list of selected markers. This prompted us to verify the presence and identity of POU4 family genes in the chicken genome. We found two genes that correspond to the mammalian *Pou4f1/Brn3a* and *Pou4f2/Brn3b*, and an orthologue of amphibian *Pou4f1.2* which was not found in mammals (Additional file 9: Figure S5A). Analysis of the number of reads mapping to the *POU4F1* and *POU4F1.2* loci (see Material and Methods) showed that the two genes collectively are expressed at higher levels in the somatosensory than in the viscerosensory ganglia, as shown previously by in situ hybridization [14, 37] (Additional file 9: Figure S5B).

GO analysis of the panel of high-confidence marker genes as a whole demonstrated significant enrichments for categories associated with the extracellular compartment, which may be a reflection of signalling processes, and with transcription factors (Fig. 4B; Additional file 10). There were also significant enrichments in terms associated with blood vessel development, likely to reflect the known overlap between mechanisms regulating blood vessel and nerve guidance [38] (Fig. 4B).

### Validation and expression pattern of selected markers

**Fig. 5:**
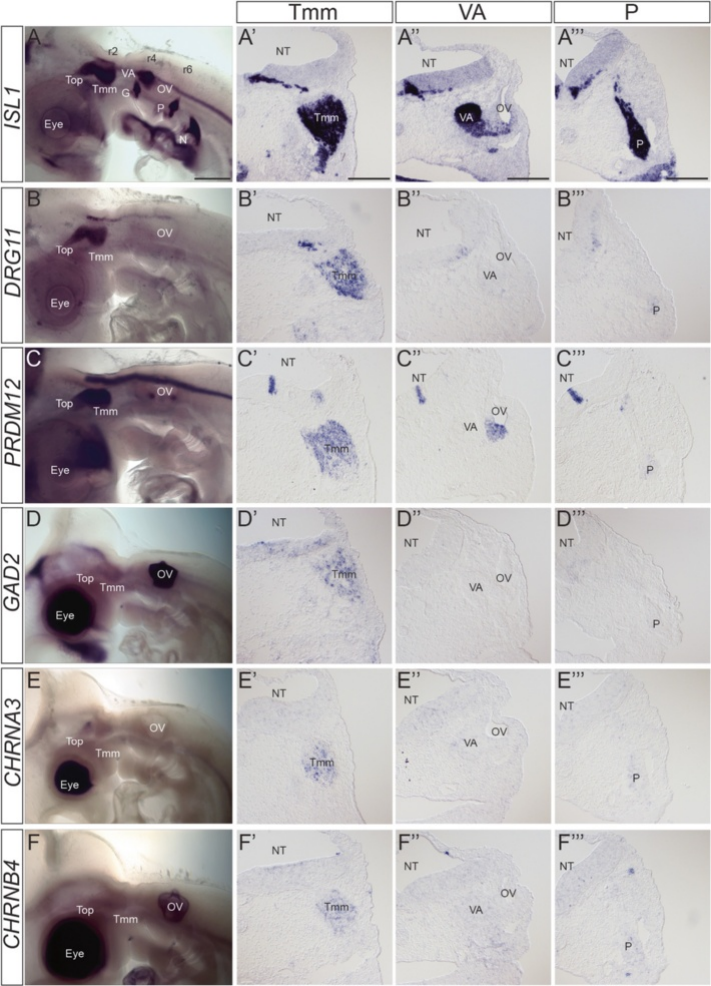
Expression patterns of trigeminal ganglion-specific markers. A) Wholemount in situ hybridization of *ISL1*at HH21 to provide anatomical localisation of all cranial sensory ganglia. A’-A”’) Transverse sections stained with *ISL1*at HH21 to provide comparative sections for other markers at the level of Tmm ganglion (A’); VA ganglion (A”); and P ganglion (A”’). B-F) Wholemount in situ hybridization of trigeminal ganglion specific markers at HH21. B’-F”’) Transverse sections at the level of the Tmm ganglion (B’-F’), VA ganglion (B”-F”) and P ganglion (B”’-F”’) stained with the named marker at HH21, showing Tmm-specific expression. B-B”’) *DRG11*; C-C”’) *PRDM12*; D-D”’) *GAD2*; E-E”’) *CHRNA3*; F-F”’) *CHRNB4*. Levels of staining in the trigeminal ganglion vary, but are stronger when compared with other ganglia. Staining can be seen in the NT although specific localisation and level varies. The dark staining in the eye and OV in D-F was not observed on sections and likely represents background. Abbreviations: G: geniculate ganglion; N: nodose ganglion; NT: neural tube; OV: otic vesicle; P: petrosal ganglion; r: rhombomere; Top: trigeminal ophthalmic lobe; Tmm: trigeminal maxillomandibular lobe; VA: vestibulo-acoustic ganglion. Scalebars: A-F: 500 μm; A’-F”’: 150 μm

**Fig. 6:**
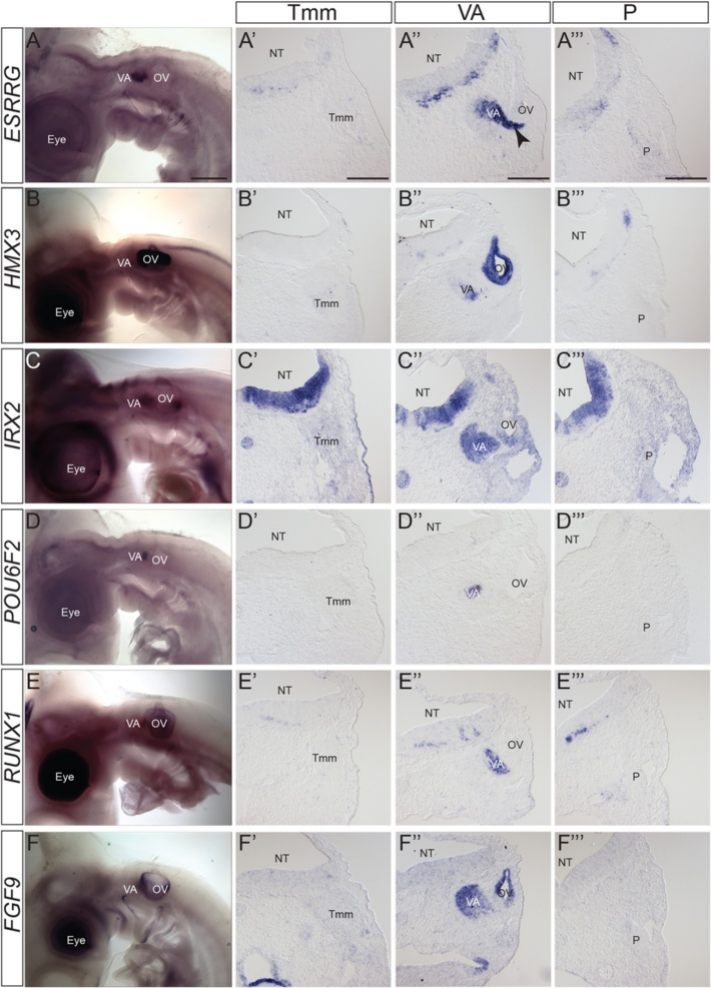
Expression patterns of vestibulo-acoustic ganglion-specific markers. A-F) Wholemount in situ hybridization of vestibulo-acoustic ganglion specific markers at HH21. A’-F”’) Transverse sections at the level of the Tmm ganglion (A’-F’), VA ganglion (A”-F”) and P ganglion (A”’-F”’) stained with the named marker at HH21, showing VA-specific expression. A-A”’) *ESRRG*; B-B”’) HMX3; C-C”’) *IRX2*; D-D”’) *POU6F2*; E-E”’) *RUNX1*; F-F”’) *FGF9*. Levels of staining in the vestibulo-acoustic ganglion vary, but are stronger when compared with other ganglia. In *ESRRG* staining is also seen in nascent neurons migrating from the OV (*arrowhead*). Staining can be seen in the NT although specific localisation and level varies. The dark staining in the eye and OV in B and E was not observed on sections and likely represents background. Abbreviations: NT: neural tube; OV: otic vesicle; P: petrosal ganglion; Tmm: trigeminal maxillomandibular lobe; VA: vestibulo-acoustic ganglion; arrowhead: nascent neurons migrating from OV. Scalebars: A-I: 500 μm; A’-I”’: 150 μm

**Fig. 7:**
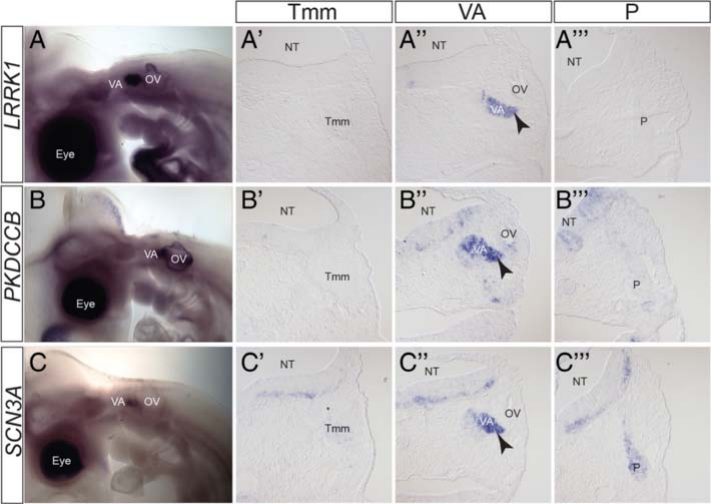
Expression patterns of vestibulo-acoustic ganglion-specific markers. A-C) Wholemount in situ hybridization of vestibulo-acoustic ganglion specific markers at HH21. A’-C”’) Transverse sections at the level of the Tmm ganglion (A’-C’), VA ganglion (A”-C”) and P ganglion (A”’-C”’) stained with the named marker at HH21, showing VA-specific expression. A-A”’) *LRRK1*; B-B”’) *PKDCCB*; C-C”’) *SCN3A*. Levels of staining in the vestibulo-acoustic ganglion vary, but are stronger when compared with other ganglia. In *LRRK1*, *PKDCCB* and *SCN3A* staining is also seen in nascent neurons migrating from the OV (*arrowhead*). Staining can be seen in the NT although specific localisation and level varies. The dark staining in the eye and OV in A-C was not observed on sections and likely represents background. Abbreviations: NT: neural tube; OV: otic vesicle; P: petrosal ganglion; Tmm: trigeminal maxillomandibular lobe; VA: vestibulo-acoustic ganglion; arrowhead: nascent neurons migrating from OV. Scalebars: A-I: 500 μm; A’-I”’: 150 μm

**Fig. 8:**
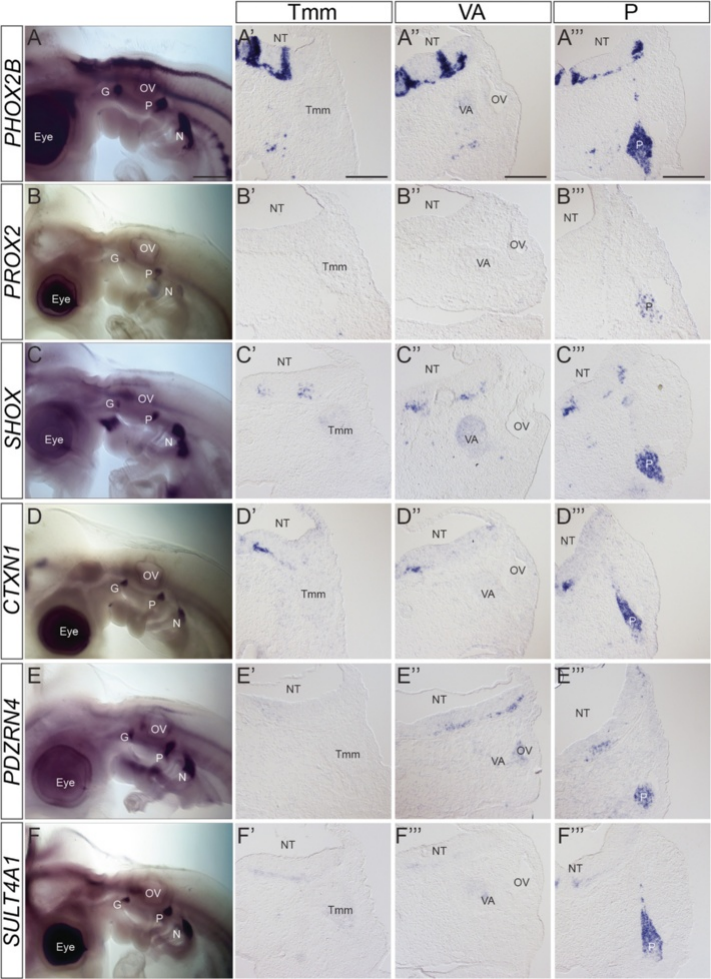
Expression patterns of epibranchial ganglia-specific markers. A-F) Wholemount in situ hybridization of epibranchial ganglia-specific markers at HH21. A’-F”’) Transverse sections at the level of the Tmm ganglion (A’-F’), VA ganglion (A”-F”) and P ganglion (A”’-F”’) stained with the named marker at HH21, showing P-specific expression. A-A”’) *PHOX2B*; B-B”’) *PROX2*; C-C”’) *SHOX*; D-D”’) *CTXN1*; E-E”’) *PDZRN4*; F-F”’) *SULT4A1*. Levels of staining in the epibranchial ganglia vary, but are stronger when compared with other ganglia. Staining can be seen in the NT although specific localisation and level varies. The dark staining in the eye and OV in A, B, D and F was not observed on sections and likely represents background. Abbreviations: G: geniculate ganglion; N: nodose ganglion; NT: neural tube; OV: otic vesicle; P: petrosal ganglion; Tmm: trigeminal maxillomandibular lobe; VA: vestibulo-acoustic ganglion. Scalebars: A-F: 500 μm; A’-F”’: 150 μm

Rather than validate gene expression for each grouping of ganglia, we chose to focus on the groupings that gave us markers of distinct sensory modalities, selecting genes representative of trigeminal for general somatosensory, vestibulo-acoustic for special somatosensory, and nodose/petrosal for viscerosensory. From candidates for these ganglia, genes with the highest expression levels and fold change of differential expression were selected for in situ hybridization analysis in wholemount and on sections of chicken embryos at stage HH21 (Figs. 5, 6, 7 and 8). Priority was given to transcription factors because they are most likely to regulate the acquisition of sensory phenotype. We recognise that our validation was not comprehensive and that it does not exclude the possibility that other genes in the panel are equally good or even better markers.

### Trigeminal ganglion-specific markers

The localisation of the cranial sensory ganglia in the HH21 chick can be clearly visualised by in situ hybridisation with *ISL1,* a marker of specific neuronal subsets including sensory neurons, which we include to allow comparison with expression in all ganglia (Fig. 5A). The trigeminal ganglion with two lobes, maxillomandibular and ophthalmic, lies at the level of the anterior hindbrain and in cross-section the ganglion can be seen adjacent to rhombomere (r)2 (Fig. 5A, A’). The transcription factor-encoding gene *DRG11* (also known as *DRGX*, *PRRXL1*) was our positive control for the trigeminal ganglion [30, 32, 39, 40] (Fig. 5B-B”’).

In the category of transcriptional regulators we analysed expression of *PRDM12* (PR homology domain-containing member 12). *PRDM12*, which is essential for human pain perception, and is required for sensory neuron development in mouse and Xenopus [41–43], showed strong trigeminal expression, with little to no staining in the other cranial sensory ganglia (Fig. 5C-C”’).

As markers with a potential link to adult neuronal function we analysed expression of *GAD2* (GABA synthetic enzyme GAD65) and *CHRNA3* and *CHRNB4* (nicotinic acetylcholine receptor subunits alpha3 and beta4). *GAD2* expression in the PNS has been described in DRG (chick) and trigeminal ganglia (rat), and *Gad2* knockout mice are sensitised to pain [44–46]. Neuronal nicotinic receptors composed of α3β4 subunits that are more restricted in expression than other subtypes, are present and show specific functions in the trigeminal ganglion of rat [47–49]. Our in situ hybridisation analysis showed *GAD2*, *CHRNA3* and *CHRNB4* staining in the trigeminal ganglion (Fig. 5D-F”’), which, in section, was weaker and in fewer neurons than *DRG11* and *PRDM12* (Fig. 5B’-F’).

Many of the trigeminal markers were expressed elsewhere in the embryo, but importantly, expression was not seen in the other cranial sensory ganglia (Fig. 5; Table 1). We focus here on hindbrain expression as it is relevant when considering potential sensory circuits. Many of the markers showed expression at the level of r2, the entry point in the hindbrain for trigeminal axons, yet the anatomical extent of staining varied. *PRDM12* was observed in a domain of strong staining in the ventral hindbrain extending from r2 into the spinal cord, and a domain of weaker staining restricted to dorsal r2 (Fig. 5C). *DRG11* was detected in a distinct domain in dorsal r2 extending caudally to the otic vesicle (Fig. 5B). *GAD2* expression was seen to extend anteriorly into r1, but with no distinct domain in r2 (Fig. 5D). Expression of *CHRNA3* and *CHRNB4* was not observed in the hindbrain (Fig. 5E, F).

**Table 1:** Summary of selected marker genes expression in the head as assessed by in situ hybridization

		Cranial sensory ganglia						Other PNS sites
		Top	Tmm	VA	G	P	N	
Trigeminal	*DRG11*	+++	+++	-	-	-	-	
	*PRDM12*	+++	+++	-	+ (distal)	+ (distal)	+ (distal)	Olfactory epithelium, ciliary ganglion, otic macular patches
	*GAD2*	++	++	-	-	-	-	
	*CHRNB4*	+	+	-	-	-	-	
	*CHRNA3*	+	+	-	-	-	-	
	*TOX2*	(+)	(+)	-	-	-	-	
	*OTX2*	(+)	(+)	-	-	-	-	Ventral otic vesicle
Vestibulo-acoustic	*ESSRG*	-	-	+++	-	-	-	
	*PKDCCB*	-	-	+++	-	-	-	Otic macular patches
	*LRRK1*	+ (distal)	+ (distal)	+++	-	-	-	
	*POU6F2*	+ (distal)	+ (distal)	+++	-	-	-	
	*RUNX1*	-	-	++	-	-	-	
	*IRX2*	-	-	++	-	-	-	Otic vesicle
	*FGF9*	-	-	++	-	-	-	
	*SCN3A*	+	+	+++	+	+	+	Terminal nerve ganglion
	*HMX3*	-	-	+	-	-	-	Dorsal otic vesicle
Epibranchial	*PHOX2B*	-	-	-	+++	+++	+++	Ciliary ganglion
	*PROX2*	-	-	-	++	++	++	Lens
	*CTXN1*	-	-	-	+++	+++	+++	
	*SULT4A1*	+ (distal)	+ (distal)	+	+++	+++	+++	Ciliary ganglion, terminal nerve ganglion
	*PDZRN4*	-	-	-	++	++	++	
	*SHOX2*	-	-	+	+++	+++	+++	
	*LHX4*	-	-	-	-	(+)	(+)	
	*MECOM*	-	-	-	-	-	-	
	*PRRX1*	-	-	-	-	-	-	Medial otic vesicle
	*PRRX2*	-	-	-	-	-	-	

Expression levels at stage HH21 are given for the cranial sensory ganglia (Top trigeminal ophthalmic, Tmm trigeminal maxillomandibular, VA vestibulo-acoustic, G geniculate, P petrosal, N nodose), as well as other sites in the PNS

### Vestibulo-acoustic ganglion-specific markers

The vestibulo-acoustic ganglion can be seen clearly with *ISL1* expression, located anterior-medial to the otic vesicle (Fig. 5A) In cross-section the vestibulo-acoustic ganglion is localised between the anterior otic vesicle and r4/r5 of the hindbrain (Fig. 5A”).

There was not a strong positive control gene for the vestibulo-acoustic ganglion because a molecular distinction between special and general somatosensory has not been described previously. However, two transcriptional regulators, *ESRRG* (estrogen-related receptor gamma) and *HMX3* (H6 family homeobox 3; also known as *NKX5.1*), have demonstrated roles in the development of inner ear structures and are important for hearing in both mice and humans [50–55]. Here our analysis showed expression of *HMX3* in the ventromedial vestibulo-acoustic ganglion and *ESRRG* in the dorsolateral vestibulo-acoustic ganglion, as well as in nascent neuronal cells migrating into the vestibulo-acoustic ganglion from the otic vesicle (Fig. 6A-B”’).

In the category of transcriptional regulators, we analysed expression of *IRX2* (Iroquois homeobox gene family member 2); *POU6F2* (POU domain, class 6, transcription factor 2; also known as *RPF1*) and, *RUNX1* (*runt*-related transcription factor 1; also known as *AML1*, *Cbfa2*). Expression patterns of *IRX2* and *POU6F2* have been described in the developing chick and mouse but no role has been reported in the vestibulo-acoustic ganglion [56–59]. In mouse *RUNX1* has a role in the development of the vestibulo-acoustic ganglion [60] but is also expressed in TrkA+ nociceptive sensory neurons of the head and trunk, including a scattered population in the trigeminal ganglion [60, 61]. In our analysis *IRX2* expression was seen in the whole vestibulo-acoustic ganglion as well as the ventral otic vesicle corresponding to the location of vestibulo-acoustic ganglion neuron production (Fig. 6C”). *POU6F2* and *RUNX1* were expressed specifically in sub-divisions of the dorsolateral vestibulo-acoustic ganglion (Fig. 6D”, E”).

As markers with a potential link to signalling we analysed expression of the signalling molecule *FGF9*, and two kinases: *LRRK1* (leucine-rich repeat kinase 1) and *PKDCCB* (protein kinase domain containing, cytoplasmic b; ENSGALG00000011166: a paralogue of PKDCC also known as VLK). *FGF9* is important for development of aspects of the inner ear including the cochlear sensory cells (also known as hair cells), but weak expression has also been reported in the mouse cochlear/acoustic ganglion (part of the vestibulo-acoustic ganglion complex) [62, 63]. Neither *LRRK1* nor *PKDCCB* has previously been linked to the vestibulo-acoustic complex [64–67]. Our analysis showed expression of FGF9 in the whole vestibulo-acoustic ganglion and *LRRK* and *PKDCCB* in the dorsolateral vestibulo-acoustic ganglion (Fig. 6F-F” and Fig. 7A-B”).

As a marker linked to adult neuronal function we analysed expression of *SCN3A* (voltage gated sodium channel type 3, alpha subunit) which in humans lies within a chromosomal locus associated with hearing loss. Expression of *SCN3A* has not been characterised in the vestibulo-acoustic ganglion in humans or mice [68]. Our analysis showed expression in the dorsolateral vestibulo-acoustic ganglion (Fig. 7C, C”).

In addition to the vestibulo-acoustic ganglion, all of the analysed genes showed expression elsewhere in the embryo (Figs. 6 and 7; Table 1). *SCN3A* and *LRRK1* showed weak staining in other cranial sensory ganglia, but were significantly stronger in the vestibulo-acoustic ganglion. Within the hindbrain the extent of expression varied. Analysis of sections at r4, the site of vestibulo-acoustic ganglion axon entry, showed *ESRRG, POU6F2*, *PKDCCB,* and *SCN3A* staining in a similar pattern from dorsal to ventral (Fig. 6A”, D” and Fig. 7B”, C”). *HMX3* and *RUNX1* were expressed in discrete domains in dorsal r4 and in ventral regions extending beyond r4 (Fig. 6B”, E”). *LRRK1* signal was found in a restricted ventral domain (Fig. 7A”) while *IRX2* and *FGF9* staining was broadly distributed throughout the r4 neuroepithelium (Fig. 6C”, F”).

### Epibranchial ganglia-specific markers

The petrosal and nodose ganglia represent the epibranchial series of cranial sensory ganglia which can be identified clearly in the HH21 *ISL1* stained embryo (Fig. 5A). In cross-section, we focused on the petrosal ganglion located near the pharyngeal endoderm, at a distance from r6/7 of the hindbrain (Fig. 5A”’). The transcription factor *PHOX2B* represented the positive control for the epibranchial ganglia [21, 33–35] (Fig. 8A, A”’).

We analysed expression of two transcription factors-encoding genes: *PROX2* (*prospero*-related homeobox gene family, member 2) and *SHOX* (short stature homeobox transcription factor). *PROX2* expression has been described in cranial sensory ganglia in zebrafish and more specifically in the epibranchial ganglia in mouse [69, 70]. *SHOX,* important for growth in humans and zebrafish, is absent in mouse where instead the related gene *Shox2* is required for long bone growth [71–73]. Roles for mouse *Shox2* in neuronal development have been shown, and expression reported in cranial sensory ganglia [74, 75]. Our analysis showed specific expression of *PROX2* and *SHOX* in the epibranchial ganglia (Fig. 8B-C”’). In cross section the staining for these markers was scattered throughout the ganglion, suggesting that these genes’ expression may differentiate subsets of neurons (Fig. 8B”’, C”’).

Other genes which we identified as good markers of the epibranchial ganglia have not been well studied at either the expression or functional level. *CTXN1* (*Cortexin 1*), encodes a single trans-membrane domain protein identified in mouse and rat cortex [76]. *PDZRN4* (PDZ and Ring domain-containing family member 4; also known as *LNX4* (ligand of Numb protein-X)) was identified *in silico* and remains largely uncharacterised [77–79]. *SULT4A1* is a member of the sulfotransferase family, cytosolic enzymes proposed to play roles in the modulation of certain neurotransmitters, and is expressed in the human and rat brain [80, 81]. Our analysis showed expression in the epibranchial ganglia (Fig. 8D-F). As for *PROX2* and *SHOX*, the proportion of cells stained in the petrosal ganglion varied with each marker (Fig. 8E”’, F”’).

All of these genes showed expression elsewhere in the embryo, with the most restricted being *PROX2* (Fig. 8; Table 1). *SULT4A1* showed weak staining in the other cranial sensory ganglia, but was significantly stronger in the epibranchial ganglia (Fig. 8F’-F”’). In the hindbrain our analysis focused on r6, the site of entry for petrosal axons. *SHOX* expression resembled that of *PHOX2B*, with discrete staining in dorsal and ventral domains, and a small number of stained cells extending between these two domains (Fig. 8A”’, C”’). *PDZRN4* also showed distinct dorsal and ventral domains of staining (Fig. 8E”’), while *CTXN1* or *SULT4A1* expression was restricted to a very small ventral domain (Fig. 8D”’, F”’).

The expression patterns of 20 out of 26 tested markers validated the differential expression in cranial sensory ganglia revealed by RNA-seq (Table 1). For each group there were exceptions where the in situ hybridisation analysis did not match expectations. These fell into two categories: i) genes which showed no, or very low, in situ signal where expected (*LHX4*, *OTX2, TOX2*) (Additional file 11: Figure S6A-C); and ii) genes which showed high in situ signal in surrounding tissue but not in the ganglion (*MECOM*, *PRRX1* and *PRRX2*) (Additional file 11: Figure S6D-F).

## Discussion and conclusions

It has long been recognised that the cranial sensory ganglia represent distinct sensory functions. Nevertheless, analysis of mechanisms underlying the development of specific cranial sensory modalities has been limited by the paucity of markers for the different cell populations. To overcome this restriction we set out to identify molecular signatures for the distinct cranial sensory ganglia at early stages of neuronal differentiation. Here we report the generation of ganglion-specific expression profiles using transcriptome-wide analysis of placode-derived neurons from isolated cranial sensory ganglia in the developing chicken embryo. Differential expression analysis of the resultant data set showed differences in the profiles that correlate with distinct functions and sensory modalities. Principal component analysis revealed three separate clusters, capturing the segregation of the cranial sensory ganglia into viscerosensory epibranchial ganglia (nodose/petrosal); general somatosensory trigeminal ganglia; and special somatosensory ganglia (vestibulo-acoustic ganglion). Our study was not designed to assess the distinction between cranial and trunk sensory neurons. Using stringent selection criteria, we report a total of 134 marker genes specific for particular cranial ganglia or groups of ganglia, and show validation of 20/26 by in situ hybridization. Of our panel of marker genes, around 20 were known to have either described expression in sensory neurons, or a link to dysfunction of the relevant sensory system. However, 113 were entirely novel, with no previously described sensory neuron-specific expression. Importantly, we identify and validate several marker genes that differentiate between the trigeminal and vestibulo-acoustic neurons, providing the first molecular signature for the distinction between embryonic “general” and “special” somatosensory neurons. Our validation was restricted to a single stage (HH 21) in a single species (chick), and the observed differential expression might be a consequence of temporal differences in the onset of expression. However, we expect the markers to be generally valid across stages and vertebrate species.

The range of genes encompassed by our panel included transcriptional regulators, components of signalling pathways and ion channels (Fig. 4; Additional file 8). As a resource this panel of ganglion-specific markers will enable us to analyse experiments in greater depth to further our understanding of the acquisition of the respective sensory modality. They will be important in unambiguously determining the fate adopted by cells when extracellular signals or transcriptional regulators are modulated in experimental settings, for example in protocols aimed to derive specific sensory neurons from human pluripotent stem cells [82].

At an individual gene level, it will be interesting to investigate their roles in producing or maintaining sensory phenotype through knockdown and ectopic expression in the future. It is possible that transcription factors play a role as regulators of cell type identity, acting in concert with known genes such as *BRN3A* and *PHOX2B* [11, 83]. We also note that several ligands and receptors, all of which have a demonstrated role in cell migration and/or axon guidance, were expressed differentially between the classes of cranial sensory ganglia, such as the netrin family gene *NTNG1* and the Robo ligand *SLIT3* in nodose/petrosal viscerosensory neurons, or the chemokine receptor *CXCR4* in trigeminal (ophthalmic/maxillo-mandibular) and vestibulo-acoustic somatosensory neurons [84–86].

The ganglion-specific expression was readily apparent for the majority of the markers tested. Within each group we found cases which further showed expression in the target region for ganglionic projections in the hindbrain. For example, we showed expression of *ESSRG* and *POU6F2* in the dorsal-most domain specifically at r4-5 level, where vestibulo-acoustic ganglion afferents enter the brainstem. Such co-ordinate gene expression in both sensory neurons and their target central neurons has been shown to be important for correct connectivity in both head and trunk [11, 39, 87]. Thus, our data supports the idea of a “sensory type code” aligning sensory neurons with central neurons of the same circuit. This might have functional consequences in the establishment of specific viscero- and somatosensory circuits and/or represent ancient evolutionary relationships.

Genome-wide analysis of gene expression considered pooled populations of neurons from each cranial ganglion, revealing groups of viscerosensory, general somatosensory and special somatosensory neurons. Our analysis of expression patterns by in situ hybridisation demonstrated that, as established in the trunk somatosensory population [2, 3], there are further subsets within these populations. It is recognised that the vestibulo-acoustic ganglion represents a complex of two smaller ganglia individually containing neurons involved in balance and hearing [88]. Interestingly, comparison in transverse section of all vestibulo-acoustic ganglion markers showed that they occupied different regions (Figs. 6 and 7). This may be due to expression in specific subgroups of neurons such as vestibular neurons versus auditory neurons, known to occupy separate regions within the vestibulo-acoustic ganglion complex in mouse [88]. Further analysis would have to be performed to determine the allocation to specific functions. Many of the trigeminal and epibranchial ganglia markers exhibited scattered expression rather than homogeneous staining throughout the ganglion as observed for *ISL1* or *PHOX2B* (Figs. 5, 6, 7 and 8). Thus, our markers highlight further complexity in the specific subtypes of sensory neuron.

The broad distinction between viscero- and somatosensory neurons has been more widely studied. The viscerosensory system is important in controlling the body’s internal milieu including many autonomic reflexes such as baroreflex regulation of the cardiovascular system [89], hypoxia regulation of the ventilatory response [90] and nutrient-induced inhibition of food-intake [91]. The general somatosensory system is involved in response to external stimuli: in rodents the importance of touch from the whiskers can be seen in the somatotopy of the whisker barrels in the cerebral cortex [92]; in humans trigeminal involvement in pain sensation can become problematic leading to migraine and trigeminal neuralgia [93]. To date the molecular fingerprint used to recognise developing viscerosensory neurons is *Phox2a* + *Phox2b* + *Ret* + *Brn3a*– *Drg11*– *Runx1*–, while that for somatosensory neurons is *Phox2a*– *Phox2b*– *Ret*– *Brn3a* + *Drg11*+ *Runx1*+. Using this limited marker set, a seminal study found that *Phox2b* acts as a regulatory switch between the two phenotypes: in *Phox2b* mutant mice, the neurons of the epibranchial ganglia up regulate *Brn3a* leading to expression of *Drg11* and *Runx1*, and hence to the conclusion that they become somatosensory [11]. It would be interesting to re-examine this situation more closely using our data set to determine how complete this transition is, and whether the resulting somatosensory cells are more similar to trigeminal or vestibulo-acoustic neurons.

Analysis in *Brn3a* mutant mice showed changes in the subtype of somatosensory neuron specified within the trigeminal ganglion, but did not examine whether the neurons switched to a viscerosensory phenotype [83]. The studies did however, show a de-repression of genes interpreted as non-neuronal [94]. Interestingly using our panel of markers for specific cranial sensory neuronal populations, we can now identify some of these genes as markers of the viscerosensory ganglia (e.g., *ANGPTL1*; *PROX2*) or special somatosensory ganglia (e.g., *IRX2*) [94].

A complementary approach to address the generation of different cranial sensory neuron phenotypes has been to focus on the embryonic origins of the cranial sensory ganglia. There are distinct neurogenic placodes for each cranial sensory ganglion, each of which has an individual developmental profile [95, 96]. Many studies have built up our understanding of patterning the specific neurogenic placodes within the cranial ectoderm across species [97]. However, while this describes the mechanisms underlying the generation of the neurogenic placodes, it does not address how or why progenitors located in different placodes acquire distinct neuronal phenotypes. It will be interesting to interfere with aspects of neurogenic placode development, for example by transplantation or treatment with specific signalling molecules [7, 9, 10], and to use our panel of molecular markers to determine the effects on viscero- versus somatosensory neuronal differentiation.

The organisation and function of the cranial sensory ganglia is highly conserved across vertebrates. The origin of the cranial sensory ganglia from neurogenic placodes also attracts considerable interest from an evolutionary perspective. Historically neurogenic placodes have, together with neural crest cells, been suggested to be vertebrate innovations, enabling the transition to a predatory lifestyle [98], and their presence in other organisms has been examined [96, 97, 99, 100]. As with development, many of the studies to date focus on the evolution of neurogenic placodes rather than their derived sensory neurons. Provided homologous genes can be found in other organisms, our molecular profiles will be invaluable in considering the evolutionary origin of the distinct sensory neuronal phenotypes [96].

## Methods

### Embryonic dissection

Fertilized chicken eggs (Winter Egg Farm, UK) were incubated in a humidified chamber at 38 °C to the correct Hamburger and Hamilton (HH) stage of development [22]. To compensate for differences in the timing of migration and neuronal differentiation/maturation in the different ganglia relative to the age of the embryo, trigeminal maxillomandibular and ophthalmic and vestibulo-acoustic ganglia were dissected at HH18, while the nodose and petrosal were dissected at HH23 [7, 21]. These stages also take into account that neural crest-derived neurons differentiate at later stages in the respective ganglia [6, 8]. The cranial nerve ganglia were dissected in L15 medium (Gibco) using electrolytically sharpened 0.125 mm tungsten wire, nerve processes and mesenchyme were removed and pooled ganglia kept on ice in L15 for 0–3 h until dissociation. For each ganglion type, the left and right ganglia of 40–60 embryos were pooled. All three replicates of the different ganglia were collected independently from new individuals.

### Electroporations and immunostaining on sections

Eggs were windowed at HH12 and pCAβ-EGFPm5 vector electroporated into the neural crest on one side of the embryo (*n* = 4) using an Electro Square Porator ECM 830 (BTX.Inc) by applying 5 pulses (6 V, 25 ms) at 1 s intervals. Eggs were sealed and incubated until HH18. The embryos were fixed in 4 % paraformaldehyde in phosphate buffered saline (PBS) for 2 h on ice, kept in 30 % sucrose in PBS for 3 h at 4 °C and embedded in OCT compound (Andwin Scientific) for cryo-sectioning. 10 μm sections were stained with anti-NFM (clone RMO270, Invitrogen, 1:5000) primary and Alexa596 anti-mouse (Life Technologies) secondary antibodies.

### Antibody staining and FACS

Cells were dissociated in trypsin-EDTA (Gibco) for 5 min at 37 °C followed by inactivation with defined trypsin inhibitors (Gibco) and trituration with a 200 μl pipette tip. At all subsequent steps cells were pelleted in protein LoBind tubes (Eppendorf) at 500xg for 1 min at 4 °C and otherwise kept on ice. Live cells were treated with Near-IR fixable live/dead stain (Invitrogen) in PBS for 5 min according to the manufacturer’s instructions. Cells were fixed for 10–15 min in 200 μl 4 % paraformaldehyde made in MOPS buffered saline (MBS) containing 0.1 M MOPS pH7.4, 1 mM EGTA, 2 mM MgSO_4_ and 125 mM NaCl. Cells were permeabilized in permeabilization buffer (PB) containing 2 % BSA, 0.1 % saponin, 5 mM DTT and 100U/ml RNAse inhibitor (Roche) in MBS. Remnants of paraformaldehyde were inactivated by addition of glycine pH 8.0 to a final concentration of 100 mM. Cells were subsequently incubated 10–15 min on ice in anti-NFM in PB (clone RMO270, Invitrogen, 1:5000). The primary antibody was replaced by anti-mouse Alexa488 secondary antibody in PB (Invitrogen, 1:1000) and cells were incubated 10 min on ice. Cells were washed briefly in MOPS-RNasin-DTT-BSA (MRDB) buffer containing 2 % BSA, 5 mM DTT and 100U/ml RNAse inhibitor (Roche) in MBS, re-suspended in 70 μl RNA-later (Ambion) and kept overnight at 4 °C. Cells were diluted by addition of 250 ml MRDB, pelleted and resuspended in 400 μl MRDB. Cells were sorted on a MoFlo Astrios sorter. Gates were set using a control sample from HH23 limb bud treated in the same way as the cranial nerve ganglia samples. The control limb sample included 50 % of cells killed by a 5 min 60 °C heat shock to set the gate for live/dead staining. Cells were sorted into 1.5 ml protein low-bind tubes containing 70 μl MRDB chilled to 4 °C during sorting. The cells were pelleted in MRDB and lyzed in RLT lysis buffer (Qiagen) containing 1 % 2-mercaptoethanol.

### RNA extraction and sequencing

Total RNA was extracted using an RNeasy micro kit (Qiagen) using the modified protocol for fixed cells as per the manufacturer’s instructions. Genomic DNA was eliminated by an on-column DNase-I treatment. RNA integrity was assessed using the Experion system (Biorad) and RNA quantity was measured using Qubit high-sensitivity kit. Libraries were prepared using Illumina mRNA-seq kit incorporating poly-(A) selection without further amplification and 100 bp paired-end reads were sequenced on an Illumina Hi-seq 2000 platform at the High-Throughput Genomics unit at the Wellcome Trust Centre for Human Genomics, Oxford, UK. Three biological replicates of each sample were generated and assigned to different lanes, with 5 samples multiplexed on each lane. The raw reads have been uploaded to the Short Read Archive (SRA, Accession number SRP068496).

### Read mapping, quantification, and differential expression

Read quality was assessed using FASTQC. rRNA reads were identified by mapping the raw reads to chicken rRNA sequences (accession numbers: Galga 18S rRNA HQ873432.1; Galga 28S rRNA EF552813.1; Galga 5S rRNA NR046276.1; Galga 5.8S rRNA DQ018753.1) and removed from the dataset. After removal of the identified rRNA, unprocessed reads were aligned to the galGal4 chicken genome assembly (Ensembl Release 71) using the splice-aware sequence aligner Gsnap [101]. MISO [102] was used to determine an average expected insert size of 116 ± 72 bp. These values were then applied in a subsequent Gsnap iteration for more accurate paired alignment. HTseq count [103] was used on the most conservative setting (intersection strict) to count reads mapped to gene models, which were determined using the Ensembl galGal4 reference genome, version 71. Normalization was performed with DEseq [26] and EdgeR [104]. Differential expression analysis was performed with both DESeq and EdgeR, with differentially expressed genes defined as only those returned by both of these methods of analysis, with an FDR (q-value) of 0.05 or below.

### Selection of markers

Prior to estimating the gene expression of ganglia-specific markers, duplicated read pairs were identified using Picard Tools’ Mark Duplicates function. Markers were selected from this pool of differentially expressed genes through the following criteria: 1) expression levels 1.5-fold higher in all samples of the high-group than all samples in the low-group; 2) minimum RPKM of 10 in the high-group; and 3) q-value < 0.05 in both DESeq and EdgeR analyses.

### Functional annotation

Gene Ontology (GO) term enrichment analyses were implemented through DAVID [105]. This analysis was applied to the differentially expressed genes identified for each ganglion and groups of ganglia in turn, against a background of all expressed genes, where expressed genes are defined as those where at least one transcript of that gene had a minimum of 80 % coverage. The threshold for significance was set as below 0.05 for the Benjamini and Hochberg q-value returned by DAVID.

### Analysis of POU4F1

Since no gene model for POU4F1 was included in the genome version used, reads mapped as described to the GENBANK model XM_003640558.2 were counted using HTseq with the same settings as for the Ensembl gene models as described above.

Pou4 family protein sequences were collected from GENBANK using a BLASTp approach and aligned using MAFFT with default settings. The alignment was trimmed manually in BioEdit [106]. A maximum likelihood tree was built using MEGA version 5.2 [107]. The Jones-Taylor-Thornton (JTT) amino acid substitution matrix was used and 150 bootstrap iterations were used to obtain support values at each node.

### Cloning

Chick cDNA from HH18-24 heads was used for PCR cloning of the markers tested. See primers below. Products were cloned into pGEMT (Promega) or pCRII (Invitrogen).

**Table:** 

Gene name	Forward primer	Reverse primer
*CHRNA3*	ATGTGACCTGGATACCCCCA	CTTCATCACTGGTCGGCCTT
*CHRNB4*	AGTGTGAACGAACGAGAGCA	ACAGGTAGGCTGGGAGTCTT
*CTXN1*	GAGCTCTCGGTCTGCACAG	CATCCCTGCCCTCTACACCA
*ESRRG*	TCTGACGGACAGCATCAACC	AGGGTTCAGGTACGGGCTAT
*FGF9*	TTTGCTCAGTGACCACCTGG	TCAGGGTCCACTGGTCTAGG
*GAD2*	TGGTGTTGAAAGGGCCAACT	TCCTGATGAGTTGCTGCTGG
*HMX3*	CAAGAACCTGCTCAACGGAG	CGCTTCATGTCGAAGGTGGA
*IRX2*	CAGGGTTACCTCTACCAGCC	TTGCAAGCTGATCCCTTCGT
*LHX4*	TACCTGATGGAGGACGGGAG	CTCGGAGAGGATCTGGTCGT
*LRRK1*	CCTTGCCTACCTGCACAAGA	CTGCTACGAGTCCATCCGAC
*MECOM*	AAAGCCATGGTAACCAGCCA	ATTGGATGGCGCTGGATTCT
*OTX2*	CGGGCATGGATTTGTTGCAT	GGTGGTGCATAGGGGTCAAA
*PDZRN4*	TGGCTCTGGCCAAACTAAGG	CTCCACCTCATTGGCTGTGT
*PKDCCB*	ACTGCACACTTGACTTCCCC	AGCGTGGGAACAGCTAAACA
*POU6F2*	CCGTCATCGGCAACCAGATA	CCATAGGAACTGCTGTCGCA
*PRDM12*	TGATCACGTCCGACATCCTG	TGAGTTCCCGTACCAGACCA
*PROX2*	TCCTCGACGTGCAGTTCAGC	CGCAGCTTTGAACACTTCGG
*PRRX1*	TTTCCGTGAGTCACCTGCTG	ACTGTGGGCACTTGATTCCT
*PRRX2*	CCCTCAGAGCCGGAAAAACT	CTGGTTCTGATGCAGGCTGA
*RUNX1*	AACCCAGAAACACGAGGCAA	CCCTTCTGCCTCAACCACAT
*SCN3A*	TGGCTGGGATGGCTTGTTAG	TTGGAAGGATTGGCTGCCAT
*SHOX*	CGGAAGGGATCTACGAGTGC	GCTGGAGTTCTTGCTGTTGC
*SULT4A1*	GGCTTGCTACAGGAAGTGGT	CCACCATGGATTCCAGCTGT
*TOX2*	AACCTCCCTGACCCTTCACT	CCGAAGGTAGCATTGGGGTT

Other constructs were obtained: *DRG11* from Prof. J Cohen (KCL, London), *PHOX2B* from Prof, JF Brunet (ENS, Paris).

### In situ hybridisation

Wholemount in situ hybridisation was carried out as described previously [8] with incubation at 65 °C. An RNAse incubation step (15mins, 37 °C) was included post-hybridisation. For in situ hybridization on section, embryos were fixed in 4 % paraformaldehyde in MOPS buffer (MB) containing 100 mM MOPS (pH 7.5), 1 mM EGTA and 2 mM MgSO_4_ for 12 h at 4 °C. Embryos were then treated in 15 % sucrose in MB for 10 h and 30 % sucrose in MB for 2 h, and embedded in OCT compound (Andwin Scientific). 10 μm cryosections were collected and processed for in situ hybridization as previously described [108].

## Additional files

Additional file 1: Figure S1. Analysis of NFM expression in neural crest-derived cells of the trigeminal ganglion at stage HH18. A-F) Immunostaining for NFM (magenta) on sections at the level of the trigeminal ophthalmic (Top, A-C) or trigeminal maxillomandibular (Tmm, D-F) ganglion in HH18 chicken embryos in which a GFP-expressing construct was electroporated in the dorsal midbrain-hindbrain region at stage HH12. GFP expression (green) indicates origin in the cranial neural crest. GFP-positive neural crest-derived cells are NFM-negative (C and F). Scale bar: 50 μm. (PDF 6525 kb) Additional file 2:
**Information for each of the 15 samples.** Number of embryos dissected, number of ganglia included, proportion and number of cells sorted, quantity and integrity (RIN number) of RNA extracted, sequencing depth and proportion of reads mapped and number of genes expressed are indicated for each replicate. Genes are counted as expressed if RPKM > 0.3. (XLSX 10 kb) Additional file 3:
**Normalized expression levels.** Number of mapped reads per kilobase of exon per million reads (RPKM) is given for each gene and for each of the 15 replicates. (XLSX 3748 kb) Additional file 4: Figure S2. A) Pearson coefficients of correlation between principal components 1–15 and RIN values. B) Pearson coefficients of correlation between principal components 1–15 and RNA quality. C) Pearson coefficients of correlation between principal components 1–15 and read counts. (ZIP 179 kb) Additional file 5: Figure S3. Expression levels of neuronal differentiation markers. Expression levels as measured by RNA-seq (RPKM) for the indicated gene is shown for the 5 ganglia (Top: trigeminal ophthalmic; Tmm: trigeminal maxillomandibular; VA: vestibulo-acoustic; Pet: petrosal; Nod: nodose). Error bar: standard error of mean (s.e.m). (PDF 1432 kb) Additional file 6:
**Differential expression analysis results for all pairwise comparisons between ganglia using both DESeq (i) and EdgeR (ii).** Data are available as compiled .xlsx spreadsheets (A) or individual .csv files (B). (ZIP 24942 kb) Additional file 7: Figure S4. A) Number of genes identified as differentially expressed (q < 0.05) in each ganglion and in the grouped Top/Tmm/VA ganglia by DESeq and EdgeR. B) Significant Gene Ontology enrichment (q < 0.05) for differentially expressed genes reported by both DESeq and EdgeR in each ganglion. Redundant GO terms were removed using REVIGO. The Hinton plot displays the FDR (Benjamini and Hochberg) corrected q-value. Top: trigeminal ophthalmic; Tmm: trigeminal maxillomandibular, VA: vestibulo-acoustic, P: petrosal, N: nodose. (PDF 385 kb) Additional file 8:
**Average normalized read count in ganglion of focus relative to all other ganglia, fold change and q-value (Benjamini and Hochberg) for the 134 high confidence markers computed using DESeq.** (XLSX 22 kb) Additional file 9: Figure S5. Molecular phylogenetic analysis and expression levels of POU4/BRN3 family genes. A) Maximum likelihood tree of POU4/BRN3 gene family in vertebrates. 4 paralogy groups are found across vertebrates. *POU4F1.2*, named after the *Xenopus* homologue, is present in chicken but was not found in mammals. Accession numbers are shown next to each entry and bootstrap values are shown at each node. The unique Pou4 sequence from invertebrate deuterostomes are used as an outgroup and the tree is rooted with *Lottia giantea* Pou4. Species abbreviations: Brafl: *Branchiostoma floridae*; Calmi: *Callorhynchus milii*; Chrpi: *Chrysemys picta;* Danre: *Danio rerio*; Galga: *Gallus gallus*; Homsa: *Homo sapiens*; Lotgi: *Lottia gigantea*; Sacko: Saccoglossus kowalevskii; Strpu: *Strongylocentrotus purpuratus*; Xentr: *Xenopus tropicalis*. B) Expression levels (RPKM) for chicken *POU4F1* and *POU4F1.2* across five cranial sensory ganglia assessed by RNAseq. The two genes are collectively expressed at high levels in the somatic but not visceral sensory neurons. Top: trigeminal ophthalmic; Tmm: trigeminal maxillomandibular, VA: vestibulo-acoustic, P: petrosal, N: nodose. (PDF 8575 kb) Additional file 10:
**Gene Ontology annotations for the 134 high confidence ganglion-specific markers.** (XLSX 13 kb) Additional file 11: Figure S6. Expression patterns of putative ganglia-specific markers that didn’t match expectations. In situ hybridization in wholemount and on sections at the level of the trigeminal maxillomandibular (Tmm), vestibulo-acoustic (VA) and petrosal (P) ganglia. A) *LHX4* expression was detected at relatively high levels in the ventral hindbrain but only at very low levels in the petrosal ganglion. B) *OTX2* staining can be observed at high levels in the rhombic lip but only slightly higher in the trigeminal than in the vestibulo-acoustic and petrosal ganglia. C) *TOX2* staining was only detected at very low levels in the ganglia in sections. D-F) *MECOM*, *PRRX1* and *PRRX2* expression was observed at high levels in the mesenchyme around, but not within, the cranial sensory ganglia. (TIF 8124 kb)
