# Supplementary figures and images for: Identification of molecular signatures specific for distinct cranial sensory ganglia in the developing chick
Source: Neural Dev. 2016 Jan 27;11:3. doi: 10.1186/s13064-016-0057-y (PMC4730756; doi:10.1186/s13064-016-0057-y)

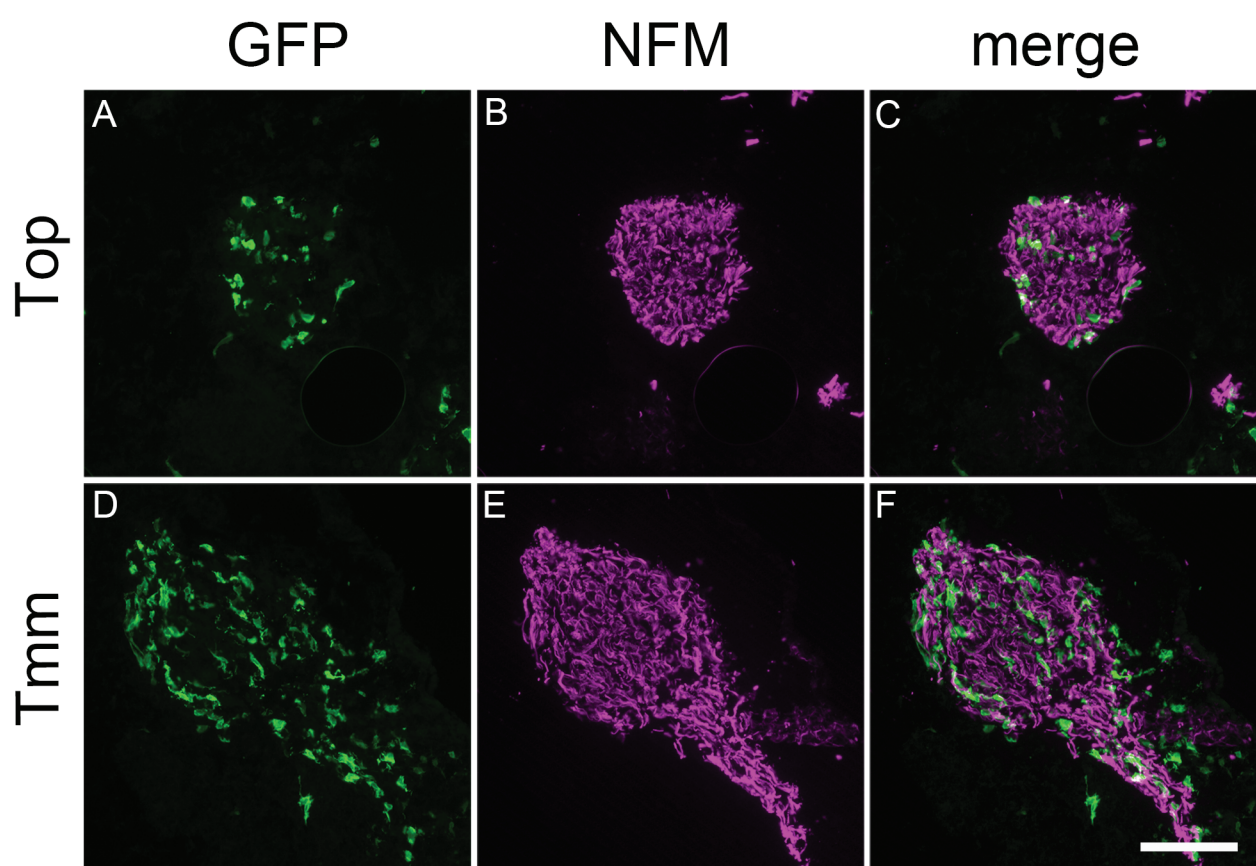

Supplement: Additional file 1: Figure S1. — Analysis of NFM expression in neural crest-derived cells of the trigeminal ganglion at stage HH18. A-F) Immunostaining for NFM (magenta) on sections at the level of the trigeminal ophthalmic (Top, A-C) or trigeminal maxillomandibular (Tmm, D-F) ganglion in HH18 chicken embryos in which a GFP-expressing construct was electroporated in the dorsal midbrain-hindbrain region at stage HH12. GFP expression (green) indicates origin in the cranial neural crest. GFP-positive neural crest-derived cells are NFM-negative (C and F). Scale bar: 50 μm. (PDF 6525 kb) [file 13064_2016_57_MOESM1_ESM.pdf]

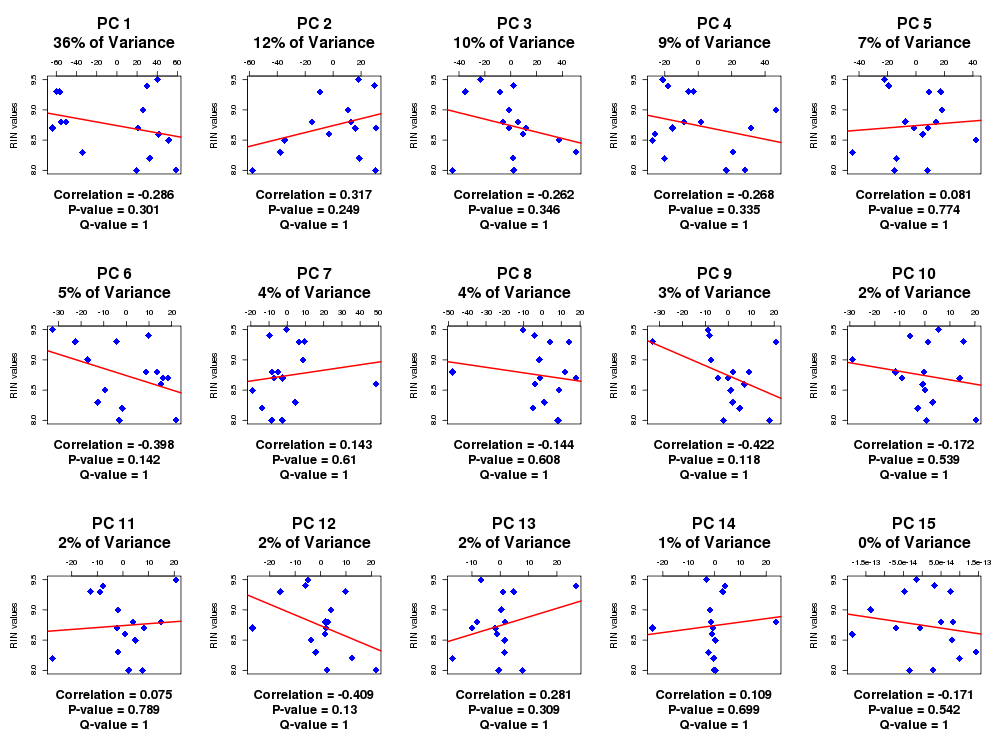

Supplement: Additional file 4: Figure S2. — A) Pearson coefficients of correlation between principal components 1–15 and RIN values. B) Pearson coefficients of correlation between principal components 1–15 and RNA quality. C) Pearson coefficients of correlation between principal components 1–15 and read counts. (ZIP 179 kb) [file 13064_2016_57_MOESM4_ESM.zip › Patthey et al Supplementary Figure 2A.png]

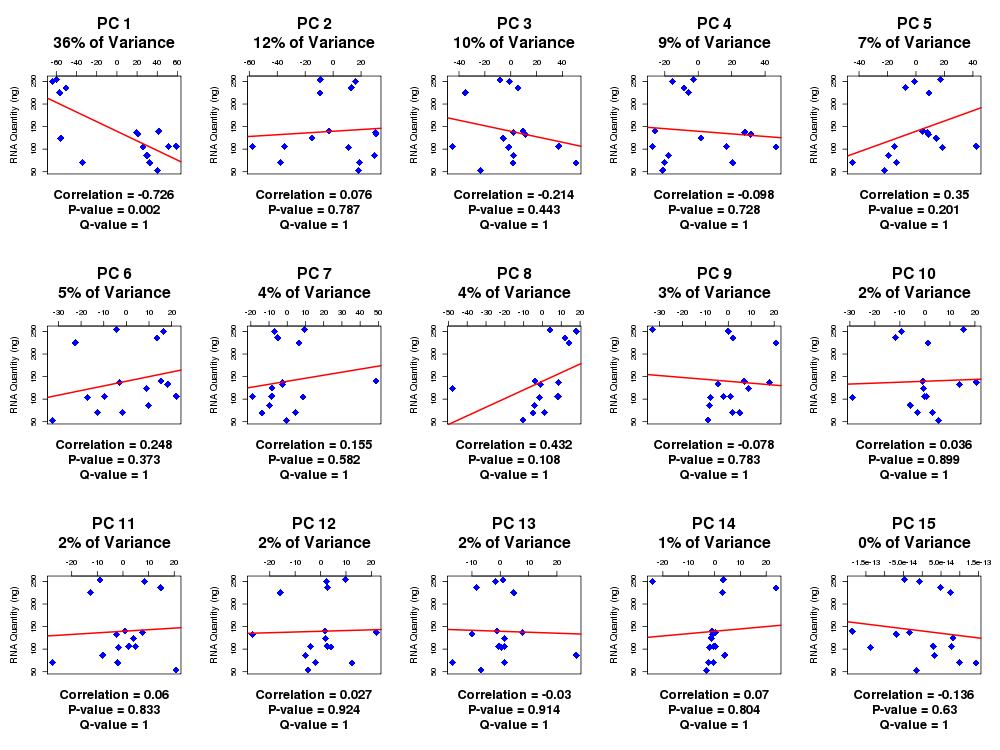

Supplement: Additional file 4: Figure S2. — A) Pearson coefficients of correlation between principal components 1–15 and RIN values. B) Pearson coefficients of correlation between principal components 1–15 and RNA quality. C) Pearson coefficients of correlation between principal components 1–15 and read counts. (ZIP 179 kb) [file 13064_2016_57_MOESM4_ESM.zip › Patthey et al Supplementary Figure 2B.png]

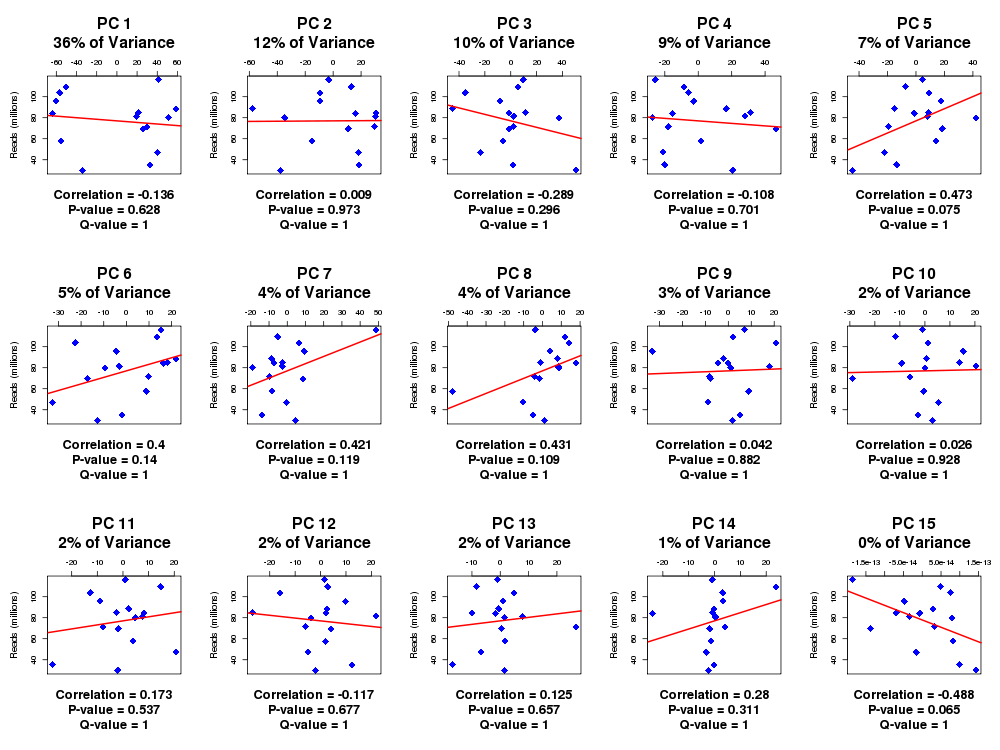

Supplement: Additional file 4: Figure S2. — A) Pearson coefficients of correlation between principal components 1–15 and RIN values. B) Pearson coefficients of correlation between principal components 1–15 and RNA quality. C) Pearson coefficients of correlation between principal components 1–15 and read counts. (ZIP 179 kb) [file 13064_2016_57_MOESM4_ESM.zip › Patthey et al Supplementary Figure 2C.png]

**A*****Elavl4***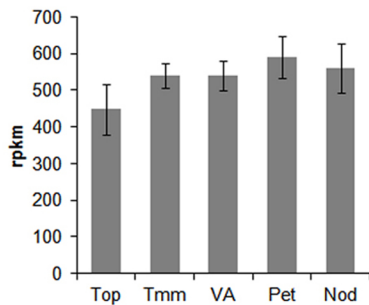**B*****Rbfox3***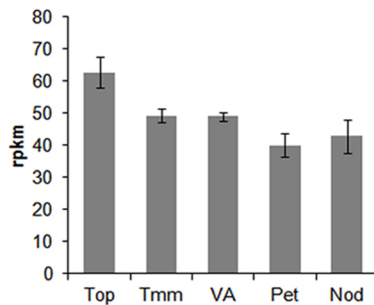**C*****Tubb3***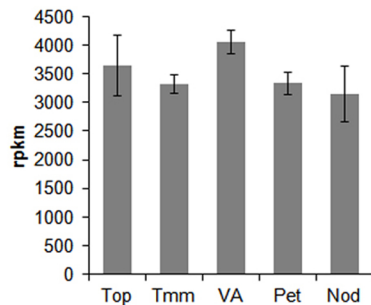**D*****Isl1***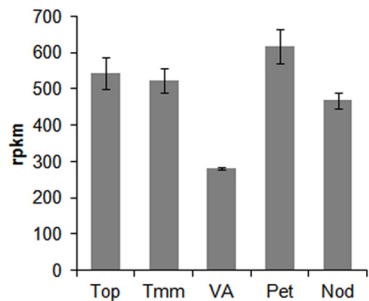**E*****Neurod1***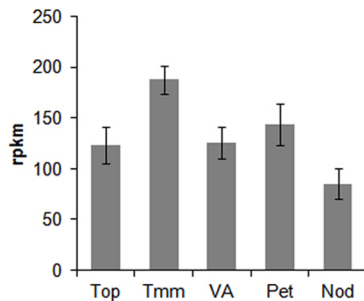**F*****Myt1***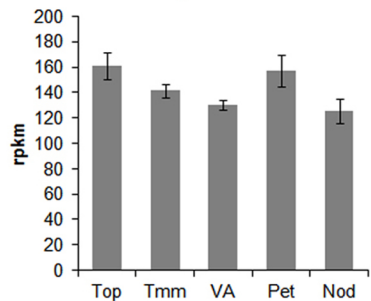

Supplement: Additional file 5: Figure S3. — Expression levels of neuronal differentiation markers. Expression levels as measured by RNA-seq (RPKM) for the indicated gene is shown for the 5 ganglia (Top: trigeminal ophthalmic; Tmm: trigeminal maxillomandibular; VA: vestibulo-acoustic; Pet: petrosal; Nod: nodose). Error bar: standard error of mean (s.e.m). (PDF 1432 kb) [file 13064_2016_57_MOESM5_ESM.pdf]

# A

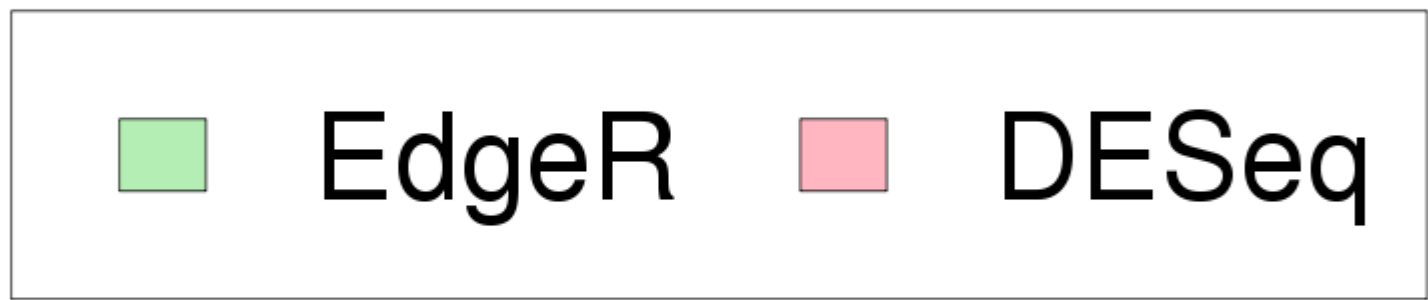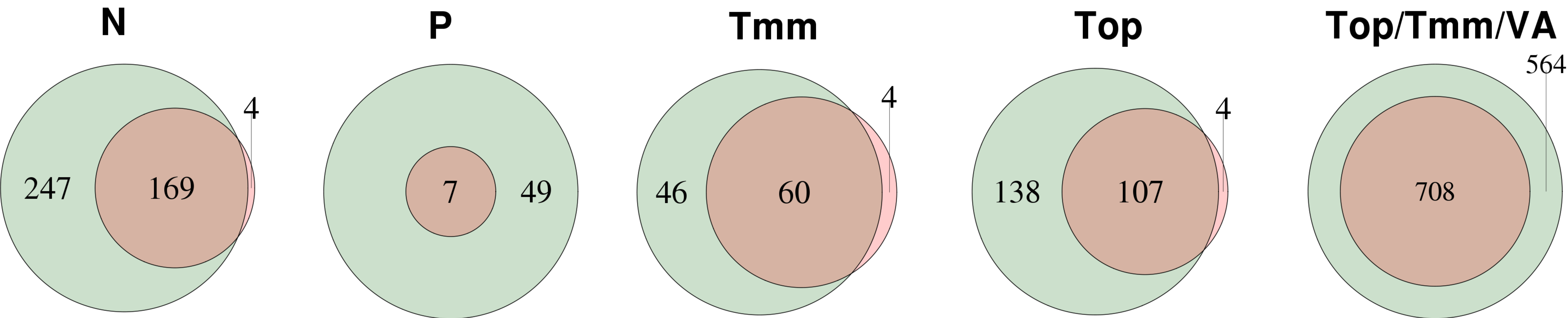

# B

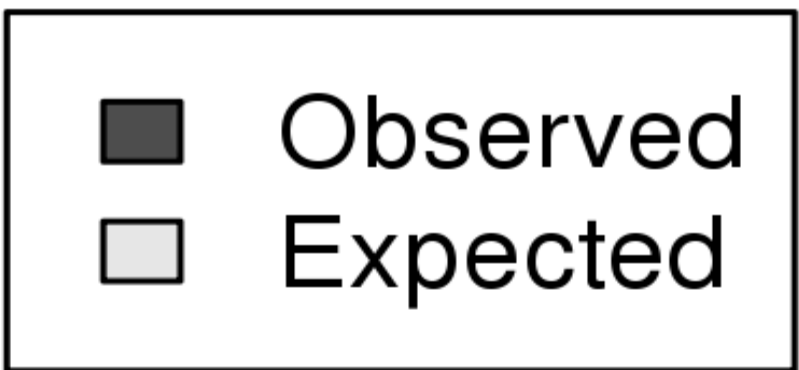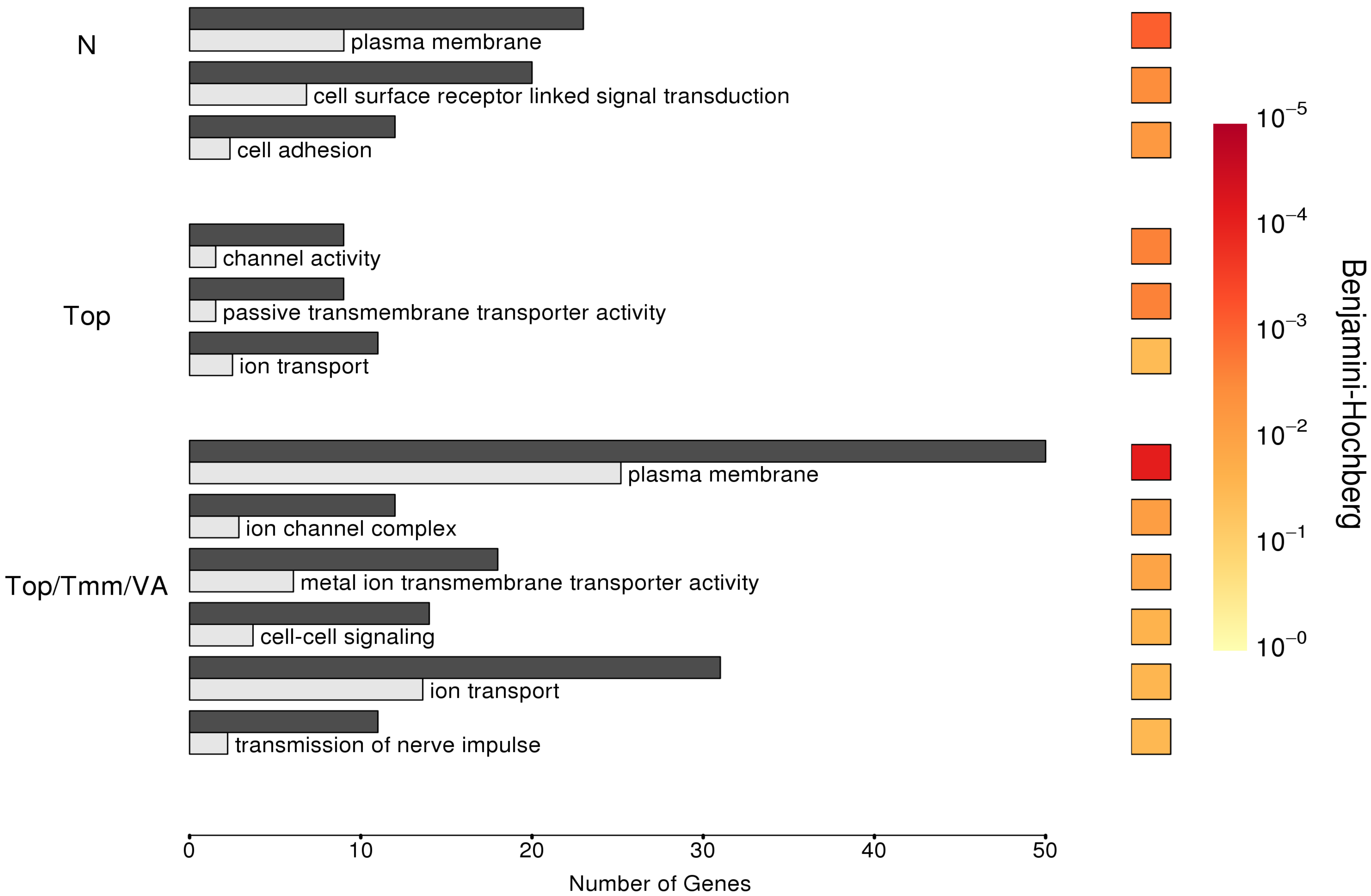

Supplement: Additional file 7: Figure S4. — A) Number of genes identified as differentially expressed (q < 0.05) in each ganglion and in the grouped Top/Tmm/VA ganglia by DESeq and EdgeR. B) Significant Gene Ontology enrichment (q < 0.05) for differentially expressed genes reported by both DESeq and EdgeR in each ganglion. Redundant GO terms were removed using REVIGO. The Hinton plot displays the FDR (Benjamini and Hochberg) corrected q-value. Top: trigeminal ophthalmic; Tmm: trigeminal maxillomandibular, VA: vestibulo-acoustic, P: petrosal, N: nodose. (PDF 385 kb) [file 13064_2016_57_MOESM7_ESM.pdf]

A

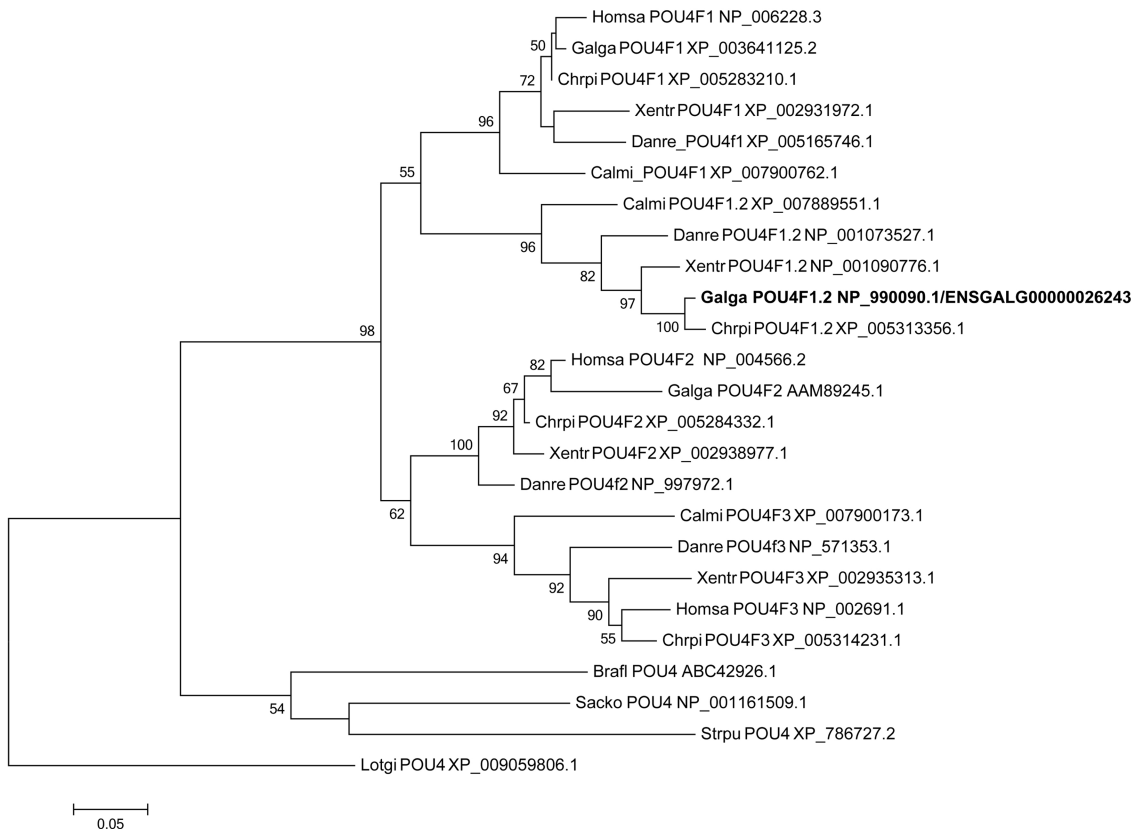

# B

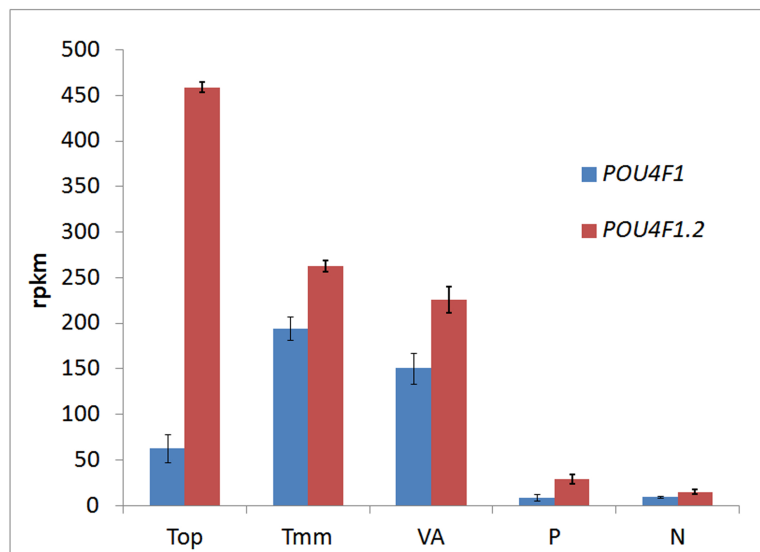

Supplement: Additional file 9: Figure S5. — Molecular phylogenetic analysis and expression levels of POU4/BRN3 family genes. A) Maximum likelihood tree of POU4/BRN3 gene family in vertebrates. 4 paralogy groups are found across vertebrates. POU4F1.2, named after the Xenopus homologue, is present in chicken but was not found in mammals. Accession numbers are shown next to each entry and bootstrap values are shown at each node. The unique Pou4 sequence from invertebrate deuterostomes are used as an outgroup and the tree is rooted with Lottia giantea Pou4. Species abbreviations: Brafl: Branchiostoma floridae; Calmi: Callorhynchus milii; Chrpi: Chrysemys picta; Danre: Danio rerio; Galga: Gallus gallus; Homsa: Homo sapiens; Lotgi: Lottia gigantea; Sacko: Saccoglossus kowalevskii; Strpu: Strongylocentrotus purpuratus; Xentr: Xenopus tropicalis. B) Expression levels (RPKM) for chicken POU4F1 and POU4F1.2 across five cranial sensory ganglia assessed by RNAseq. The two genes are collectively expressed at high levels in the somatic but not visceral sensory neurons. Top: trigeminal ophthalmic; Tmm: trigeminal maxillomandibular, VA: vestibulo-acoustic, P: petrosal, N: nodose. (PDF 8575 kb) [file 13064_2016_57_MOESM9_ESM.pdf]

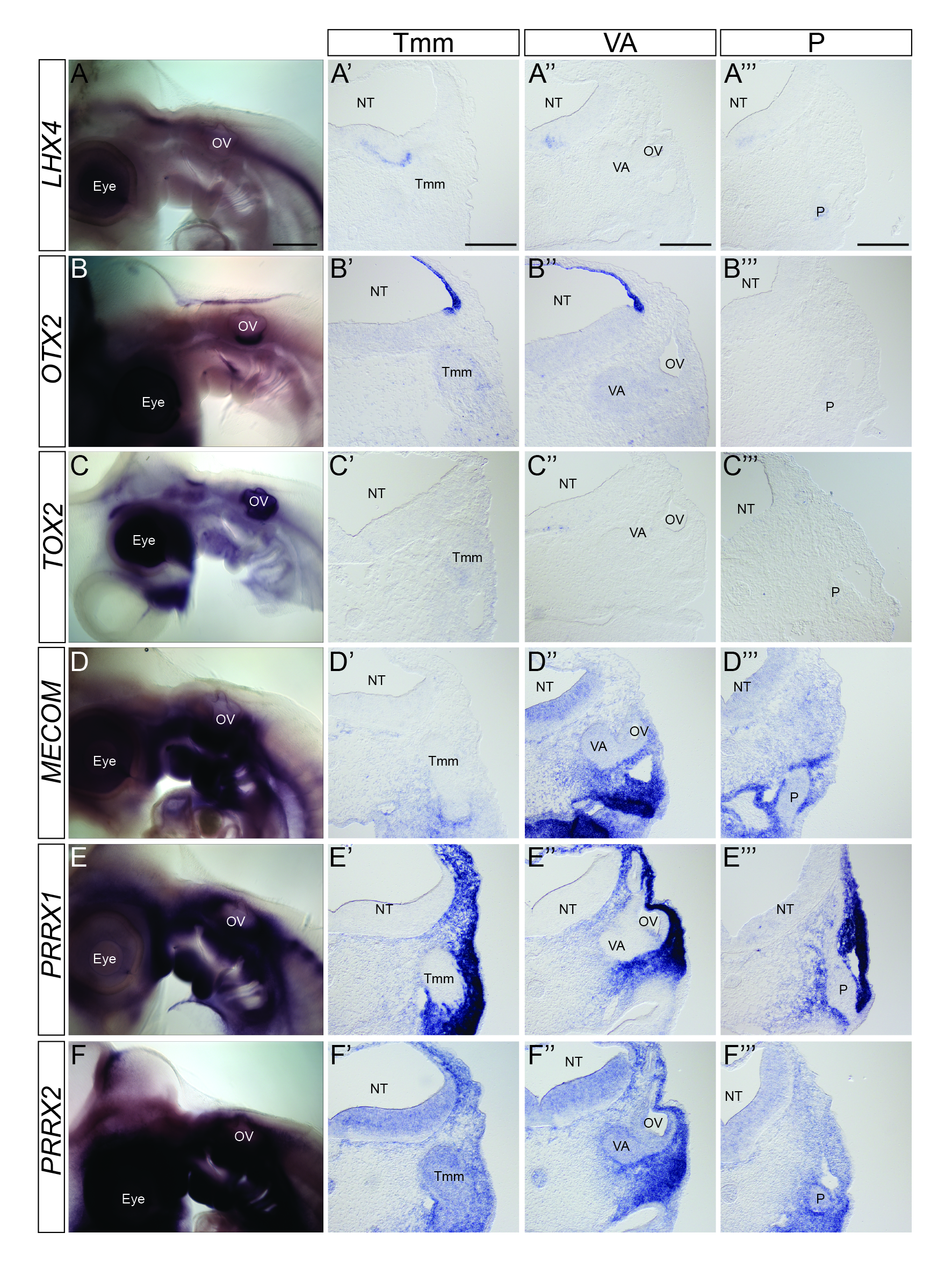

Supplement: Additional file 11: Figure S6. — Expression patterns of putative ganglia-specific markers that didn’t match expectations. In situ hybridization in wholemount and on sections at the level of the trigeminal maxillomandibular (Tmm), vestibulo-acoustic (VA) and petrosal (P) ganglia. A) LHX4 expression was detected at relatively high levels in the ventral hindbrain but only at very low levels in the petrosal ganglion. B) OTX2 staining can be observed at high levels in the rhombic lip but only slightly higher in the trigeminal than in the vestibulo-acoustic and petrosal ganglia. C) TOX2 staining was only detected at very low levels in the ganglia in sections. D-F) MECOM, PRRX1 and PRRX2 expression was observed at high levels in the mesenchyme around, but not within, the cranial sensory ganglia. (TIF 8124 kb) [file 13064_2016_57_MOESM11_ESM.tif]
